# Revealing Robust Room Temperature Ferromagnetism in Gd‐Doped Few‐Layered MoS_2_ Thin Films

**DOI:** 10.1002/advs.202510366

**Published:** 2025-08-29

**Authors:** Aswin kumar Anbalagan, Weng‐Kent Chan, Ming‐Hsuan Wu, Fang‐Chi Hu, Hsin‐Hao Chiu, Amr Sabbah, Mayur Chaudhary, Shivam Gupta, Kai‐Wei Chuang, Ashish Chhaganlal Gandhi, Ching‐Yu Chiang, Huang‐Ming Tsai, Shu‐Chih Haw, Kirankumar Venkatesan Savunthari, Hong‐Ji Lin, Li‐Chyong Chen, Kuei‐Hsien Chen, Nyan‐Hwa Tai, Yu‐Lun Chueh, Sheng Yun Wu, Hsin‐Yi Tiffany Chen, Andrew L. Walter, Chih‐Hao Lee

**Affiliations:** ^1^ Department of Engineering and System Science National Tsing Hua University Hsinchu 300044 Taiwan; ^2^ National Synchrotron Light Source II Brookhaven National Laboratory Upton NY 11973 USA; ^3^ College of Semiconductor Research National Tsing Hua University Hsinchu 300044 Taiwan; ^4^ Department of Physics National Dong Hwa University Hualien 97401 Taiwan; ^5^ Institute of Atomic and Molecular Sciences Academia Sinica Taipei 10617 Taiwan; ^6^ Center for Condensed Matter Sciences National Taiwan University Taipei 10617 Taiwan; ^7^ Department of Materials Science and Engineering National Tsing Hua University Hsinchu 300044 Taiwan; ^8^ Institute of Nuclear Engineering and Science National Tsing Hua University Hsinchu 300044 Taiwan; ^9^ Department of Electrical Engineering National Tsing Hua University Hsinchu 300044 Taiwan; ^10^ Electrical and Computer Engineering Lyle School of Engineering Southern Methodist University Dallas TX 75205 USA; ^11^ National Synchrotron Radiation Research Center Hsinchu 30076 Taiwan; ^12^ Department of Chemistry and Chemical Biology Northeastern University Boston MA 02115 USA; ^13^ Center of Atomic Initiative for New Materials National Taiwan University Taipei 10617 Taiwan

**Keywords:** defect healing mechanism, DFT, ferromagnetism, MoS_2_, rare‐earth doping

## Abstract

2D MoS_2_ holds great promise for spintronics, yet is limited by intrinsic diamagnetism. This study demonstrates inducing ferromagnetic behavior in MoS_2_ films doped with 0.47% Gd, achieving an ultrahigh saturation magnetization of 454 emu/cm^3^ in a few‐layered film over 11‐times higher than bulk films (40 nm). Raman spectroscopy, X‐ray photoelectron spectroscopy, X‐ray magnetic circular dichroism, and density functional theory (DFT) calculations reveal an interplay between Gd dopants and Mo, S vacancies (V_1Mo+2S_), leading to the formation of bound magnetic polarons (BMPs) that drive ferromagnetic ordering. H_2_S annealing and DFT calculations reveal that defect healing reduces the saturation magnetization by 83%. High sulfur migration barrier in few‐layered films helps preserve BMPs, thereby sustaining ferromagnetism, whereas lower migration barriers in bulk films lead to suppression. These findings highlight the synergy between Gd doping and defect engineering in achieving ultrahigh room‐temperature ferromagnetism, offering a scalable strategy for developing high‐performance 2D magnetic materials for spintronic applications.

## Graphical abstract

Ultrahigh room‐temperature ferromagnetism is demonstrated in few‐layer 0.47% Gd‐doped MoS2 films, with a saturation magnetization of 454 emu cm^−^^3^, 11 times higher than bulk. Experimental and DFT calculations reveal that the interplay between Gd dopants and Mo, S vacancies (V_1Mo+2S_), forms bound magnetic polarons that drive ferromagnetic ordering.

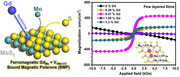

## 1 Introduction

2D Transition metal dichalcogenide (TMD) materials have attracted significant attention in the last few decades due to their excellent electronic and optical properties,^[^ ^1^, ^2^, ^3^, ^4^, ^5^ ^]^ offering potential applications in transistors,^[^ ^6^ ^]^ resistive random‐access memory,^[^ ^7^ ^]^ nanoelectronics,^[^ ^8^ ^]^ and catalysis.^[^ ^9^ ^]^ Among many 2D TMDs, molybdenum disulfide (MoS_2_) is a non‐magnetic semiconductor in its intrinsic form, and inducing magnetism into MoS_2_ is vital for developing its application in spintronics. As such, developing various strategies to induce and control magnetism is essential for integrating magnetic structures into quantum information devices. In recent years, extensive studies have been conducted to induce ferromagnetism into various 2D materials by inducing defects or doping into the MoS_2_ structure. Defect engineering is achieved by introducing structural defects into 2D TMDs by exposing them to ionizing radiations such as electrons, protons, or gamma rays.^[^ ^10^, ^11^, ^12^, ^13^ ^]^ These modifications can have beneficial and adverse effects on the targeted magnetic, electronic, and optical properties.^[^ ^14^, ^15^, ^16^, ^17^ ^]^

On the other hand, various research groups have demonstrated that doping either transition or rare‐earth elements,^[^ ^18^, ^19^, ^20^, ^21^, ^22^, ^23^, ^24^, ^25^, ^26^, ^27^, ^28^, ^29^, ^30^, ^31^, ^32^ ^]^ into the MoS_2_ structure is a simple and efficient way to induce ferromagnetism in non‐magnetic 2D TMDs. Recent work by Wang, Y., et al. used ion implantation to introduce transition metals such as Mn, Fe, Ni, and Co doping into MoS_2_ single crystals, and their resulting room temperature (RT) saturated magnetic moments are only 125, 15, 4, and 40 emu cm^−3^, respectively.^[^ ^21^ ^]^ In another work, Fu et al. achieved ferromagnetic behavior demonstrated in monolayer MoS_2_ by in situ substitutional doping of Fe atoms, reporting a magnetic moment of ≈30–35 µ emu.^[^ ^24^ ^]^ However, there are still some challenges in the current research on transition metal doping into MoS_2_, like the Curie temperature is below room temperature and the saturation magnetization (M_s_) value being relatively low.^[^ ^18^, ^19^, ^20^, ^21^, ^24^, ^25^, ^26^, ^27^, ^28^, ^29^, ^30^, ^31^ ^]^ Therefore, an alternative approach is required to achieve a higher saturation magnetization value on a uniformly deposited MoS_2_ film for large‐scale or industrial applications.

Thus, rare‐earth elements have been referred to as a strong competitor to transition metal elements since their unoccupied 4f or 5d states coupled with S 3p and Mo 4d states from the MoS_2_ are believed to achieve comparably huge magnetism. In addition to that, theoretical calculations reported by Majid, A., et al.,^[^ ^33^ ^]^ demonstrated the values of the total magnetic moment were 3.3, 8.1, 8.5, 6.8, and 6.4 µ_B_ for Sm, Eu, Gd, Tb, and Dy doped MoS_2,_ respectively. Along with theoretical predictions, Zhao, Q., et al. experimentally confirmed that Dy^[^ ^32^ ^]^ and Ho^[^ ^23^ ^]^ doped MoS_2_ has a M_s_ values of ≈0.027 and 0.055 emu g^−1^. Subsequently, anomalous large ferromagnetism was observed in the case of Nd‐doped MoS_2_ reported by Ding, X., et al.,^[^ ^22^ ^]^ where it displayed magnetism of 53 emu cm^−3^ at RT and 1640 emu cm^−3^ at 5 K. These experimental findings and first‐principles calculations on rare‐earth‐doped MoS_2_ have prompted further exploration for practical applications in spintronics devices.

To the best of our knowledge, no reported work has employed magnetron sputtering for directly doping rare‐earth elements into MoS_2_ to induce room‐temperature ferromagnetism. In this study, we utilized a magnetron co‐sputtering technique for the growth of MoS_2_ films and doping Gd into MoS_2_ to study their effects on the magnetic, structural, and chemical properties. Interestingly, we noted a magnetic phase transition and an ultrahigh ferromagnetic behavior at room temperature by doping a certain amount of Gd concentration into MoS_2_ few‐layered films. This unexpected result prompted a detailed investigation using a combination of synchrotron and laboratory‐based characterization techniques, supported by density functional theory calculations. Finally, the role of post‐annealing treatment of the Gd‐doped MoS_2_ films and the role of defects appear to be crucial and are explored in depth in the following sections.

## 2 Results and Discussion

**Figure 1:**
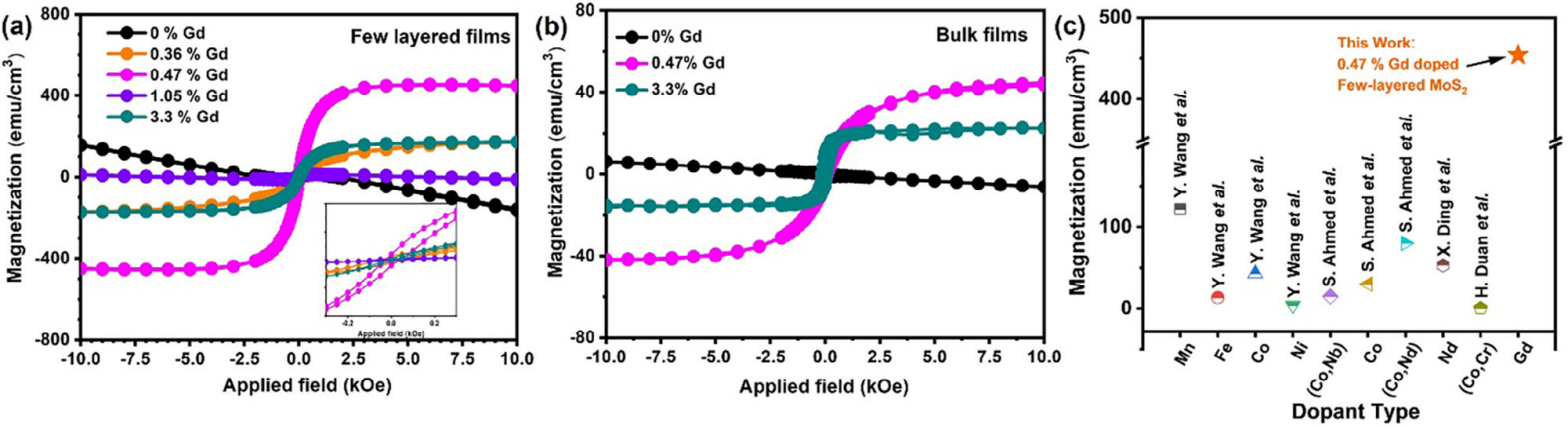
M‐H curve of: a) few‐layered (3.5 nm) Gd‐doped MoS_2_ films with different Gd dopant concentrations. And the inset shows the enlarged view. b) Bulk (40 nm) Gd‐doped MoS_2_ films with different Gd dopant concentrations and c) A graph illustrating the magnetization values generated in MoS_2_‐based TMDs through various dopants, as reported in previous studies,^[^ ^19^, ^20^, ^21^, ^22^, ^34^ ^]^ compared with the findings of this present work.

**Figure** **1**a,b shows the in‐plane field‐dependent magnetization measurements (M‐H) of pristine and Gd‐doped MoS_2_ films, including few‐layered (3.5 nm) and bulk (40 nm) samples, measured at room temperature (RT), after removing all magnetic background contributions. A detailed explanation of the various background removal processes is provided in our previous work.^[^ ^11^ ^]^ Pristine MoS_2_ films remained diamagnetic; however, as the films started doping with Gd atoms, the samples exhibited ferromagnetic behavior at RT. The M_s_ values of few‐layered (3.5 nm) films are 172, 454.4, 14.5, 181.6 emu cm^−3^ at 0.36, 0.47, 1.05, and 3.3% of Gd doping concentration, respectively. The inset in Figure 1a shows the enlarged features of the M‐H curve of few‐layered (3.5 nm) films, demonstrating that 0.47% Gd‐doped few‐layered MoS_2_ films have higher M_s_ and coercivity values when compared to other doping concentrations. To further correlate the effects of Gd doping to the origin of ferromagnetic behavior, bulk (40 nm) MoS_2_ films at 0.47 and 3.3% Gd doping concentrations were examined. However, the M_s_ values of 0.47% and 3.3% Gd‐doped bulk MoS_2_ films are 11 times and 9 times lower compared to few‐layered (3.5 nm) Gd‐doped MoS_2_ films, respectively (Figure 1b). In addition, Figure 1c shows the comparison plot of M_s_ values obtained in this work (0.47% Gd‐doped MoS_2_ few‐layered film) with other published works.^[^ ^19^, ^20^, ^21^, ^22^, ^34^ ^]^ Table S1 (Supporting Information) also provides a comparison of the M_s_ values obtained for MoS_2_‐based TMDs through doping in this study with those reported in previous works.

To compare the magnetization induced by Gd doping in this work with the previous DFT calculations conducted by Majid, A., et al.,^[^ ^33^ ^]^ we estimated the magnetic moment present per Gd dopant based on the doping concentration. However, our experimental results indicate that the anomalous magnetization observed in few‐layered (3.5 nm) Gd‐doped MoS_2_ samples cannot be solely attributed to Gd dopants. This suggests that the ultrahigh M_s_ value could arise from various kinds of defects such as V_MoS6_, Mo_S_, V _MoS2,_ V_S_, V_S2_, V_MoS3_, S_2Mo_, Mo_S2,_ etc.,^[^ ^10^, ^11^, ^35^, ^36^, ^37^, ^38^ ^]^ or from strain induced by Gd incorporation into the MoS_2_ lattice.

**Figure 2:**
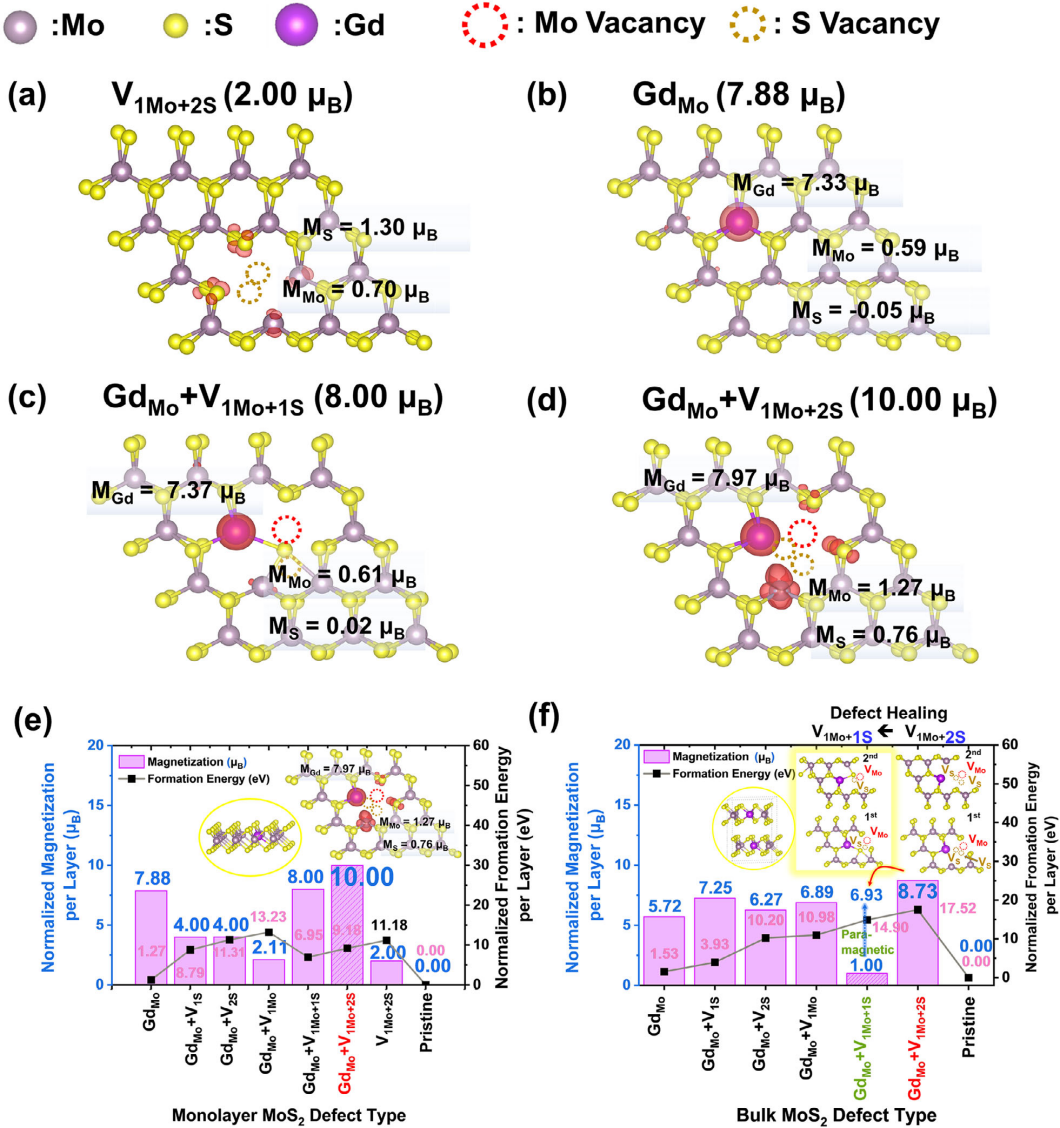
Optimized structures and spin density distribution of monolayer MoS_2_ models a) with one Mo point defect and two S point defects (V_1Mo+2S_), b) Gd substituting one Mo atom (Gd_Mo_), c) Gd_Mo_ combined with V_1Mo+1S_, and d) Gd_Mo_ combined with V_1Mo+2S_. Spin density distributions (red cloud) highlight the magnetic behavior of each structure. e) Calculated magnetic moments (in µ_B,_ marked in blue) and formation energies (in eV, marked in red) for monolayer MoS_2_ with different defect types. f) Comparison of magnetic moments and formation energies for bulk MoS_2_ with analogous defect types. Grey (Mo), yellow (S), and dark purple (Gd).

Herein, we attempted to understand the origin of ultrahigh magnetization for the experimental case of 0.47% Gd‐doped few‐layered (3.5 nm) films by DFT calculations. First, we calculated various configurations of Gd doping and combinations of Mo and S vacancies to examine their different magnetization in the MoS_2_ structure. **Figure** **2**a–d presents the optimized structures and spin density distributions for monolayer MoS_2_ models (V_1Mo+2S_, Gd_Mo_, Gd_Mo_+V_1Mo+1S_, Gd_Mo_+V_1Mo+2S_). Detailed parameters for those mentioned above and other defect structures, including those shown in Figure 2e, are presented in Figure S1 (Supporting Information). Figure 2e shows that magnetization is primarily induced by substituting a Mo atom with Gd in a configuration (Gd_Mo_) featuring a Mo vacancy and two adjacent S vacancies (V_1Mo+2S_). The Gd_Mo_+V_1Mo+2S_ configuration yields a total magnetization of 10 µ_B_, representing an enhancement compared to the Gd_Mo_ configuration without vacancies (7.88 µ_B_) following the theoretical calculations by Ouma et al.^[^ ^39^ ^]^ Other defect combinations, such as Gd_Mo_+V_1S_ (4 µ_B_), Gd_Mo_+V_2S_ (4 µ_B_), Gd_Mo_+V_1Mo_ (2.11 µ_B_), and Gd_Mo_+V_1Mo+1S_ (8.00 µ_B_), exhibit lower magnetization values. Their magnetization reduction originates from the hybridization of the density of states of Gd with MoS_2_ states near the Fermi energy, leading to spin polarization cancellation between the spin‐up and spin‐down density of states of the MoS_2_ states and the Gd dopant near the Fermi energy, as depicted in Figure S2 (Supporting Information) density of states plots. The detailed structural parameters are illustrated in Table S2 (Supporting Information). This hybridization suppresses the overall magnetization in these configurations. In contrast, the Gd_Mo_+V_1Mo+2S_ configuration demonstrates minimal hybridization between the density of states of Gd and the density of states of MoS_2_, preserving the intrinsic magnetic contribution of Gd. This lack of hybridization ensures the highest magnetization, further emphasizing the magnetic enhancement provided by the Gd_Mo_+V_1Mo+2S_ configuration. Specifically, the V_1Mo+2S_ vacancy contributes an additional 2 µ_B_, which adds directly to Gd_Mo_(7.88 µ_B_), resulting in a maximized total magnetization of 10 µ_B_ for Gd_Mo_+V_1Mo+2S_.

The monolayer MoS_2_ model showed that Gd_Mo_+V_1Mo+2S_ exhibits the highest magnetization. We extended our investigation to bulk MoS_2_ to determine whether this defect maintains high magnetization or undergoes a reduction consistent with experimental data. For bulk MoS_2_, the defect type Gd_Mo_+V_1Mo+2S_ is theoretically predicted to exhibit ferromagnetism (8.73 µ_B_ depicted in Figure 2f). However, the M‐H curve measurements, as shown in Figure 1b, reveal 11 times decrease in magnetization after 0.47% Gd doping, highlighting a significant discrepancy that requires further investigation. This discrepancy likely arises from a different defect type, Gd_Mo_+V_1Mo+1S,_ rather than Gd_Mo_+V_1Mo+2S_, as verified by DFT calculations. The defect formation energies calculated suggest that Gd_Mo_+V_1Mo+1S_ (14.90 eV) is more thermodynamically favorable to form in bulk than Gd_Mo_+V_1Mo+2S_ (17.52 eV). Gd_Mo_+V_1Mo+1S_ exhibits paramagnetic behavior, which explains the reduction of magnetization in bulk MoS_2_. Figure S3 (Supporting Information) supports the paramagnetic behavior of Gd_Mo_+V_1Mo+1S_ in bulk MoS_2_, showing that the formation energies of the antiferromagnetic (E_AFM_, where magnetization is 1.00 µ_B_) and ferromagnetic (E_FM_, where magnetization is 6.93 µ_B_) states for Gd_Mo_+V_1Mo+1S_ in the bulk sample are identical (E_AFM_ = E_FM_ = 14.90 eV, antiferromagnetism was modeled by assigning opposite magnetization directions to the primary contributors, the Gd atoms, in bulk sample). The paramagnetic nature of Gd_Mo_+V_1Mo+1S_ suggests that defect healing may have occurred in the bulk MoS_2_ after Gd sputtering, whereby sulfur vacancies are partially restored, shifting ferromagnetic Gd_Mo_+V_1Mo+2S_ into the paramagnetic Gd_Mo_+V_1Mo+1S_ configuration. The underlying mechanism of this process will be explained as follows.

**Figure 3:**
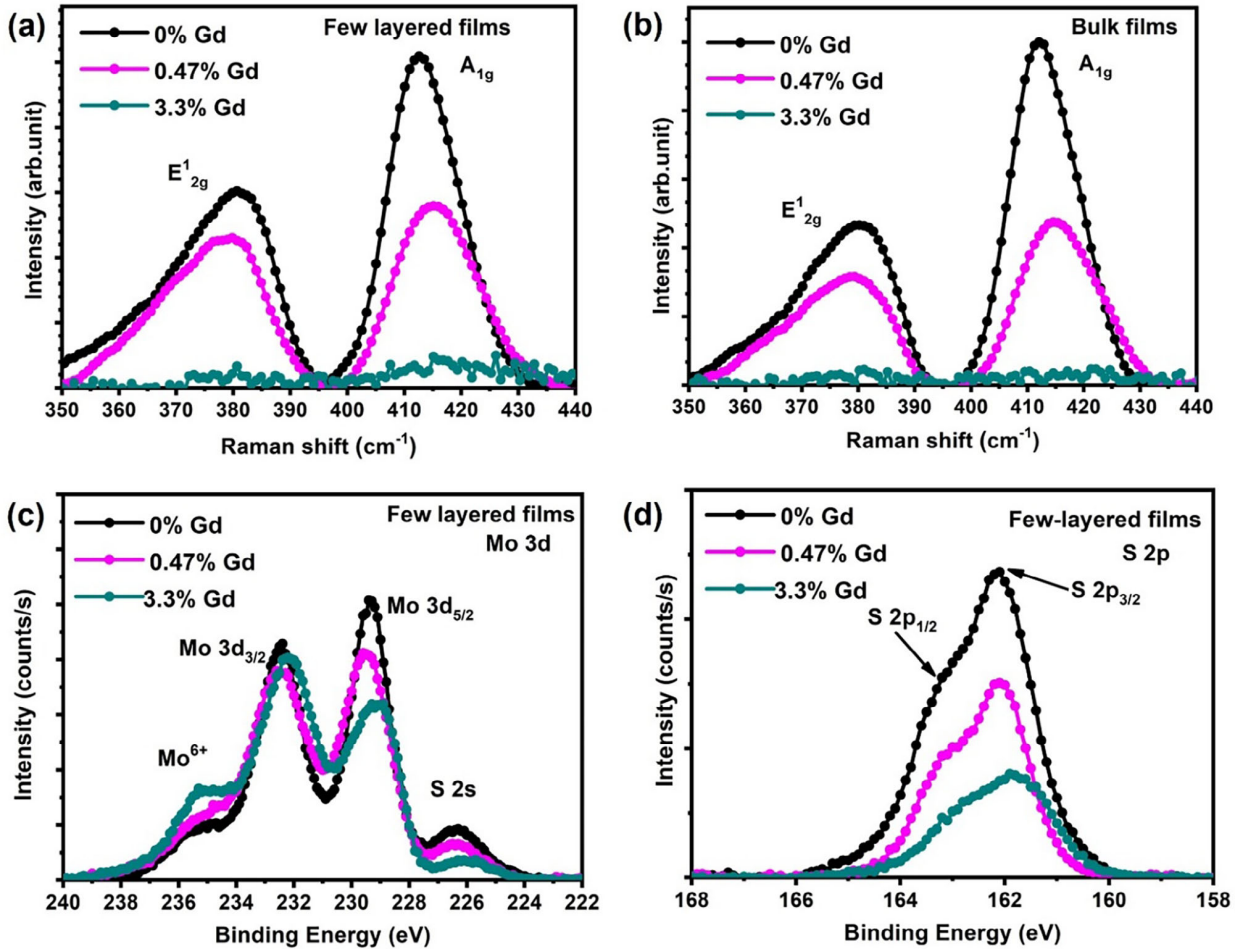
Raman spectra of a) few‐layered (3.5 nm) and b) bulk (40 nm) Gd‐doped MoS_2_ films with different Gd doping concentrations and XPS spectra of c) Mo 3d and d) S 2p of few‐layered (3.5 nm) Gd‐doped MoS_2_ films.

To confirm the presence of the hypothesized defect effect and assess the occurrence of defect healing in bulk versus few‐layered MoS_2_, we employed Raman spectroscopy, XPS, XMCD to characterize structural, chemical states, and magnetic evolutions in these films. Raman spectroscopy (**Figure** **3**a,b) was measured on MoS_2_ few‐layered (3.5 nm) and bulk (40 nm) films before and after doping to analyze changes in the chemical vibrational modes within the films. The intensities of the $E_{2g}^{1}$ and A_1g_ modes drop drastically in both few‐layered (3.5 nm) and bulk (40 nm) Gd‐doped MoS_2_ samples as the Gd dopant concentration increases, where these features almost diminish in case of 3.3% Gd‐doped MoS_2_ films. This degradation in peak intensity is likely due to the structural disorder induced by doping, leading to the bond‐breaking between Mo and S atoms. Additionally, increasing Gd dopant concentration causes a shift in the A_1g_ peak toward a higher wavenumber in both few‐layered (3.5 nm) and bulk (40 nm) films. This blue shift in the A_1g_ mode could be attributed to structural disruption and increased defects caused by Gd doping. Chakraborty et al.^[^ ^40^ ^]^ proposed that, unlike the $E_{2g}^{1}$ mode, the A_1g_ mode of MoS_2_ is sensitive to doping, with p/n‐type doping causing a blue/red shift due to strong electron‐phonon coupling. Accordingly, the moderate blue shift observed in the A_1g_ peak in both 3.5 and 40 nm thick MoS_2_ films after Gd doping can be explained by partial p‐type doping. Furthermore, as the doping concentration increases, a slight red shift is observed in the $E_{2g}^{1}$ peak, possibly due to increases in defect concentrations (V_S,_ V_2S_ and V_Mo_), consistent with the theoretical calculations by Kou et al.^[^ ^41^ ^]^

Subsequently, XPS measurements were conducted to investigate changes in the chemical states and doping concentration of the few‐layered (3.5 nm) and bulk (40 nm) MoS_2_ films before and after Gd doping. The survey spectrum (Figure S4, Supporting Information) of 3.3% Gd‐doped MoS_2_ few‐layered (3.5 nm) films confirmed the absence of contamination. Atomic concentrations of Gd, Mo, and S were calculated from the fitted peak areas of Gd 3d, Mo 3d, and S 2p spectra, normalized by their respective relative sensitivity factors (RSFs). The detailed quantification procedure and values are provided in Tables S3 and S4 (Supporting Information). The Mo 3d core‐level spectra of few‐layered (3.5 nm) and bulk (40 nm) MoS_2_ films (Figure 3c; Figure S5a, Supporting Information) revealed that pristine MoS_2_ films exhibit a doublet peak (i.e., 3d_3/2_ and 3d_5/2_) together with an S 2s peak ≈226 V, attributing to the Mo bonding with S atoms. With increasing doping concentration, the intensity of the S 2s peak decreases while Mo^6+^ features increase compared to pristine films. This indicates degradation of Mo^4+^ and S 2s states due to the bond‐breaking between Mo and S, consistent with previous studies.^[^ ^11^, ^27^, ^29^ ^]^

The S 2p core‐level XPS spectra of Gd‐doped MoS_2_ films (Figure 3d; Figure S5b, Supporting Information) exhibit doublet features (S 2p_3/2_ and S 2p_1/2_), with intensity loss after doping indicating S desorption from the MoS_2_ structure due to the bond‐breaking. Additionally, Gd 3d spectra (Figure S5c,d) confirm that Gd dopants exist predominantly in the trivalent state rather than in a metallic state, strongly suggesting the incorporation of Gd into the MoS_2_ lattice. This outcome aligns with the SQUID measurements and Raman spectroscopy, which indicate that the higher M_s_ value in 0.47% Gd‐doped MoS_2_ few‐layered films results from the combined effects of Gd doping and defects/vacancies created in the MoS_2_ films.

**Figure 4:**
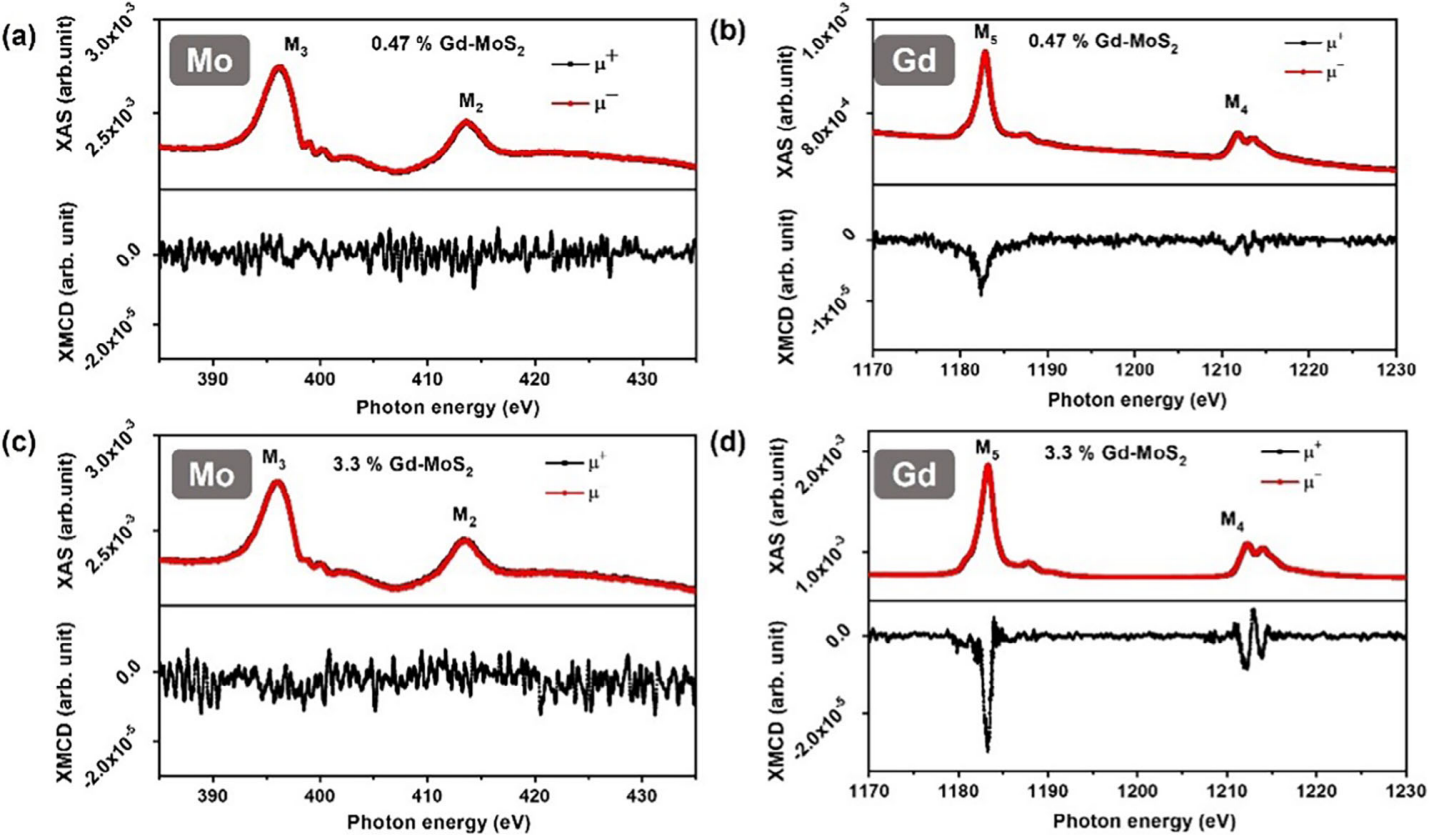
XAS & XMCD spectra of: a) Mo M2,3 edge and b) Gd M4,5 edge absorption spectra of 0.47% Gd‐doped MoS2 few‐layered (3.5 nm) films, and c) Mo M2,3 edge d) Gd M4,5 edge of 3.3% Gd‐doped MoS2 few‐layered (3.5 nm) films.

To further explore the origin of microscopic magnetic moments, XMCD measurements were performed on 0.47% and 3.3% Gd‐doped few layered MoS_2_ films (**Figure** **4**). The XMCD spectra of the Mo M_2,3_ edge (Figure 4a,c) indicate that the magnetic signal from the 3d orbital lies within the statistical error, suggesting that Mo does not significantly contribute to magnetism in Gd‐doped MoS_2_ films. In contrast, the Gd M_4,5_ edge (Figure 4b,d) shows a prominent magnetic signal originating from Gd 4f orbitals. Notably, the Gd XMCD intensity is higher in 3.3% Gd‐doped films than in 0.47% Gd‐doped MoS_2_ films. However, M‐H measurements reveal that the M_s_ value initially increases up to 0.47% Gd doping but decreases at higher doping levels. This indicates that magnetism in Gd‐doped MoS_2_ films arises from a combined effect of defects/vacancies and their interactions with Gd atoms.

Raman spectra, XPS, and XMCD analyses provide clear evidence of Mo─S bond‐breaking in both few‐layered and bulk MoS_2_ films, ruling out simpler defect configurations characterized involving isolated vacancies of a single element (V_1S_, V_2S_, or V_1Mo_). The high magnetization observed in few‐layered MoS_2_ aligns with DFT calculations for Gd_Mo_+V_1Mo+2S_ (10 µ_B_), whereas the lower magnetization in bulk corresponds to Gd_Mo_+V_1Mo+1S_ (paramagnetic). These results indicate the simultaneous presence of Gd dopant and Mo‐S vacancies (V_1Mo+2S_ or V_1Mo+1S_). These findings support the hypothesis that Gd doping and Gd‐induced defects contribute to the ferromagnetic behavior in Gd‐doped MoS_2_ films.

Furthermore, various characterization techniques, including TEM cross‐section, XRD, XRF mapping, and SEM, confirmed the thickness, structure, uniformity, and distribution of Gd doping in the MoS_2_ films (Figures S6–S9, Supporting Information). XRD analysis of the bulk (40 nm) films revealed a lattice expansion in the d‐spacing value of MoS_2_ after Gd doping. SEM morphology showed that the nano‐worm‐like structure observed in pristine bulk (40 nm) MoS_2_ becomes disrupted as the Gd doping concentration increases to 3.3%. These findings are consistent with Raman spectroscopy and XPS analyses, providing robust evidence for structural disorder and the presence of sulfur vacancies. However, due to experimental limitations, conclusive results could not be obtained for the few‐layered (3.5 nm) Gd‐doped MoS_2_ films.

**Figure 5:**
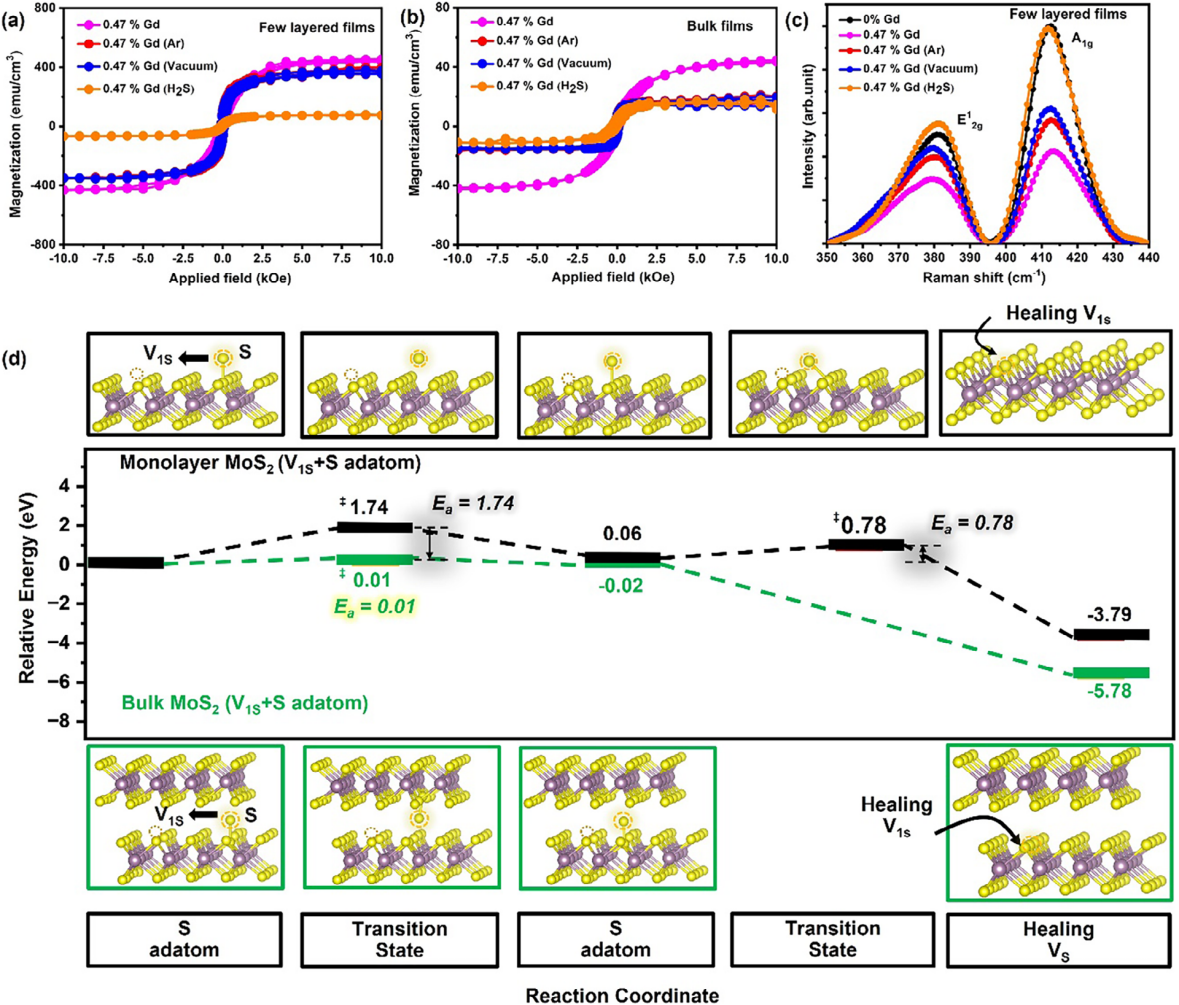
M‐H curves of 0.47% Gd‐doped MoS_2_ a) few‐layered (3.5 nm) and b) bulk (40 nm) films before and after post‐annealing in argon, vacuum, and H_2_S (15 min). Raman spectra of 0.47% Gd: MoS_2_ samples before and after post‐annealing for c) 3.5 nm films and d) Reaction coordinate diagram comparing the S defect healing process in monolayer and bulk MoS_2_. The diagram illustrates the initial states (unhealed single S vacancy(V_1S_), and an S adatom in both monolayer and bulk MoS_2_), transition states, and final states (defect healed MoS_2_ where V_1S_ are filled by S adatom). The S adatom migration energy barrier (E_a_) for monolayer MoS_2_ and S migration energy barrier for bulk MoS_2_ are indicated at each site. Atom types are depicted with grey (Mo), yellow (S), and dark purple (Gd).

To further explore the mechanism responsible for inducing ultrahigh FM behavior, post‐annealing M‐H measurements were performed on 0.47% Gd‐doped MoS_2_ films with few‐layered (3.5 nm) and bulk (40 nm) thicknesses. The films underwent post‐annealing treatments in argon, vacuum, and H_2_S environments for 15 min to facilitate sulfur defect healing. As shown in **Figure** **5**a, the M‐H measurements (in‐plane) of the few‐layered (3.5 nm) films after annealing revealed M_s_ values of 378, 366, and 77 emu cm^−^^3^ for treatments in argon, vacuum, and H_2_S, respectively. Similarly, Figure 5b shows that the bulk (40 nm) films exhibited M_s_ values of 18, 16.6, and 12.9 emu cm^−^^3^ under the same conditions. Both thicknesses showed a reduction in M_s_ values after post‐annealing, with the most pronounced decrease observed following H_2_S treatment.

Notably, the few‐layered (3.5 nm) films displayed an 83% reduction in M_s_ after H_2_S treatment, which can be attributed to a decrease in the density of magnetic polarons due to the recombination of sulfur vacancies. This result aligns with previous studies on Nd‐doped MoS_2_, where a high defect concentration was correlated with elevated M_s_ values while post‐annealing reduced ferromagnetic behavior.^[^ ^42^ ^]^ These findings provide a crucial insight into the critical role of defects/vacancies in achieving robust ferromagnetism in doped MoS_2_ films.

To further correlate the observed decrease in magnetization with the chemical structure of thin films after post‐annealing under different environments, Raman spectroscopy was performed. Figure 5c shows the Raman spectra of 0.47% Gd‐doped MoS_2_ few‐layered (3.5 nm) films before and after post‐annealing treatment under different environments. Notably, the intensity of the Raman peaks nearly doubled, approaching pristine conditions, following H_2_S post‐annealing compared to treatments under argon and vacuum. The Raman spectra revealed a redshift in the A_1g_ peak and a blueshift in the $E_{2g}^{1}$ peak for all post‐annealed films, regardless of the environment. Kou et al.^[^ ^41^ ^]^ reported that increasing concentrations of Vs and Vs_2_ defects caused minimal changes or a slight blueshift in the A_1g_ peak, while the $E_{2g}^{1}$ peak exhibited a more pronounced redshift. Similar trends in $E_{2g}^{1}$ peak shifts associated with variations in sulfur vacancy density have been reported by various groups.^[^ ^11^, ^44^ ^]^ In our films annealed under H_2_S, the blueshift in the $E_{2g}^{1}$ peak suggests a reduction in defect density, particularly in sulfur vacancies (Vs, Vs_2_). William et al.,^[45]^ using DFT calculations, demonstrated that separated sulfur vacancies caused a blueshift in the A_1g_ peak, whereas adjacent vacancies or line defects involving multiple sulfur vacancies led to redshifts. Thus, the observed redshift in the A_1g_ peak in our films likely arises from the rearrangement or reduction of defects/vacancies following post‐annealing treatment.

The combined results from M‐H measurements and Raman spectroscopy further confirm the partial structure recovery of the MoS_2_ films after post‐annealing. To differentiate how S vacancy reconfiguration proceeds in bulk versus few‐layered MoS_2_, we conducted DFT calculations to compare the S migration barrier (E_a_) on the monolayer MoS_2_ surface and within the bulk MoS_2_. The reaction coordinate diagram (Figure 5d) provided kinetic insights into S vacancy passivation. These findings clarify how Gd_Mo_+V_1Mo+2S_ or Gd_Mo_+V_1Mo+1S_ defects arise in monolayer/few‐layered and bulk MoS_2_. For monolayer MoS_2_, the S adatom migration barrier is 1.74 eV, revealing unfavorable kinetics and thus implying limited S vacancy passivation.

In contrast, bulk MoS_2_ exhibited an S migration barrier of only 0.01 eV, significantly lower than the thermal energy of 0.025 eV at RT, indicating that spontaneous vacancy reconfiguration is highly feasible. These results are consistent with previous DFT studies,^[^ ^44^ ^]^ highlighting a pronounced difference in S migration kinetics between monolayer and bulk MoS_2_. This transition from the ferromagnetic Gd_Mo_+V_1Mo+2S_ configuration to the paramagnetic Gd_Mo_+V_1Mo+1S_ state provides a comprehensive explanation for the observed reduction in magnetization in bulk samples. Meanwhile, few‐layered MoS_2_ (Gd_Mo_+V_1Mo+2S_) retains its high magnetization because the elevated 1.74 eV barrier limits S vacancy rearrangement.

**Figure 6:**
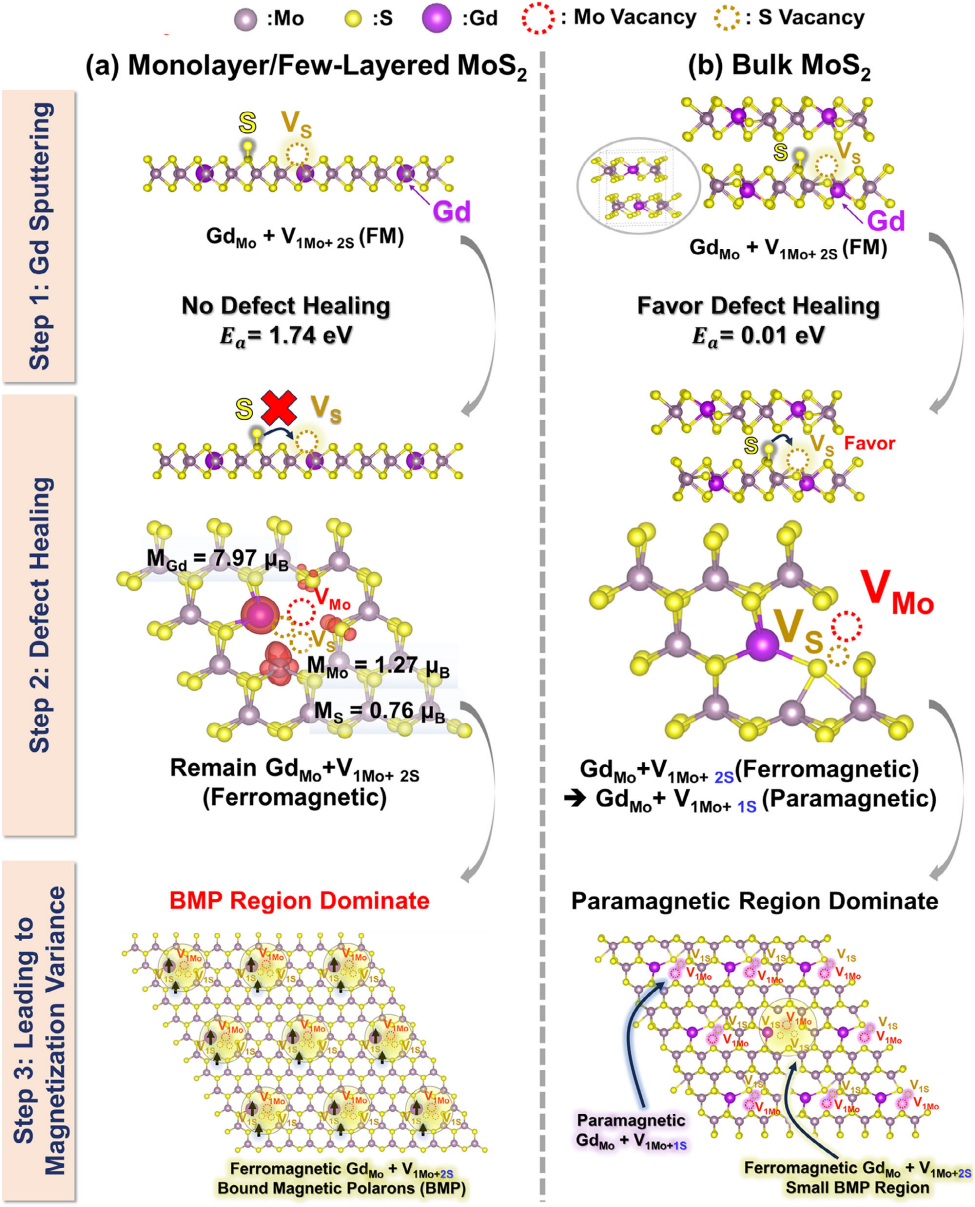
Schematic illustration of Gd‐sputtering‐induced Mo and S vacancy formation and subsequent defect healing in a) monolayer/few‐layered and b) bulk MoS_2_, leading to different magnetization. Step 1: Gd sputtering introduces S vacancies, which emerge as S adatoms in monolayer/few‐layered MoS_2_ or in bulk MoS_2_. Step 2: Defect healing varies with S migration barriers (E_a_). High barriers for S adatoms in monolayer/few‐layered MoS_2_ inhibit defect healing, preserving Gd_Mo_+V_1Mo+2S_, which leads to ferromagnetism. Lower migration barriers in bulk MoS_2_ favor defect healing, thereby shifting from ferromagnetism defect (Gd_Mo_+V_1Mo+2S_) into paramagnetic defect (Gd_Mo_+V_1Mo+1S_). Step 3: These distinct defects lead to variations in magnetization. In monolayer/few‐layered MoS_2_, Gd_Mo_+V_1Mo+2S_ defects form BMPs, resulting in a BMP‐dominated ferromagnetic state (yellow cloud). In bulk MoS_2_, defect healing predominantly produces Gd_Mo_+V_1Mo+1S_, yielding a paramagnetic state with a minor BMP region (purple cloud) from unrecovered Gd_Mo_+V_1Mo+2S_. Mo (grey), S (yellow), and Gd (dark purple).

The schematic in **Figure** **6** depicts the Gd‐sputtering‐induced S vacancy formation and subsequent defect‐healing processes in monolayer/few‐layered (Figure 6a) versus bulk MoS_2_(Figure 6b), ultimately leading to different magnetic states. In Step 1, Gd sputtering creates S vacancies, which emerge as S adatoms in monolayer/few‐layered MoS_2_ or bulk MoS_2_. During Step 2, S migration will govern the defect healing of S vacancies. A high barrier (1.74 eV) in monolayer/few‐layered MoS_2_ inhibits defect healing, preserving Gd_Mo_+V_1Mo+2S_ and resulting in ferromagnetism. In contrast, a low barrier (0.01 eV) in bulk MoS_2_ favors defect healing and facilitates the transition from ferromagnetic defect (Gd_Mo_+V_1Mo+2S_) to paramagnetic defect (Gd_Mo_+V_1Mo+1S_), causing a shift to paramagnetism. In Step 3, these distinct defects (Gd_Mo_+V_1Mo+2S_ vs Gd_Mo_+V_1Mo+1S_) determine the observed magnetization. Monolayer/few‐layered MoS_2_ contains Gd_Mo_+V_1Mo+2S_, which forms bound magnetic polarons (BMP) that couple ferromagnetically when the spacing between them remains within the optimal range. This spacing allows magnetic moments to communicate through carriers, leading to a strong ferromagnetic state. However, in bulk MoS_2_, most Gd_Mo_+V_1Mo+2S_ defects heal into Gd_Mo_+V_1Mo+1S_, suppressing BMP formation and resulting in a predominantly paramagnetic response. A small fraction of unrecovered Gd_Mo_+V_1Mo+2S_ may still contribute to minor ferromagnetism (≈40 emu cm^−3^ measured at 0.47% Gd doping, as shown in Figure 1b). This interplay between defect type and inter‐polaron distance thus governs the macroscopic magnetic response.

The significant magnetic contribution of Gd, enhanced by V_1Mo+2S_ defect, highlights the critical role of this defect configuration in achieving high magnetization. The contrasting dimensionality‐dependent (few‐layered vs bulk MoS_2_) behavior, characterized by suppressed defect healing in monolayers and facile sulfur recovery in bulk, provides crucial insights into the mechanisms underlying magnetization variations. These findings establish a foundational understanding of the interplay between defect chemistry and dimensional effects in Gd‐doped MoS_2_, offering new opportunities for designing tunable magnetic materials with tailored properties. This study confirms that the ultrahigh M_s_ value observed in few‐layered Gd‐doped MoS_2_ films arises from the additive effects of Gd dopants and sulfur vacancies. Furthermore, this work experimentally demonstrates a pathway for achieving ultrahigh FM behavior by doping rare‐earth atoms into uniformly grown MoS_2_ films.

## 3 Conclusion

In this work, we demonstrated ultrahigh room temperature ferromagnetism in 0.47% Gd‐doped MoS_2_ thin films, achieving an ultrahigh M_s_ value of 454 emu cm^−3^ in few‐layered (3.5 nm) films, 11 times higher than bulk (40 nm) films. Raman spectroscopy, XPS, XMCD, and DFT calculations reveal that this exceptional magnetization arises from the interplay between Gd dopants and Mo, S vacancies (V_1Mo+2S_), forming bound magnetic polarons (BMPs that drive ferromagnetic ordering. Post‐annealing in an H_2_S environment reduces Ms by 83%, highlighting the critical role of sulfur vacancies in maintaining ultrahigh magnetization. Further transition‐state analyses using DFT calculations indicate that defect healing is kinetically hindered in monolayer films, preserving ferromagnetic BMP configurations (Gd_Mo+_V_1Mo+2S_). In contrast, defect healing occurs more readily in bulk films, transforming ferromagnetic defects into paramagnetic states (Gd_Mo+_V_1Mo+1S_). These findings emphasize the importance of defect dynamics in tailoring magnetic properties and provide a scalable approach for inducing and tuning ferromagnetism in 2D materials. Our results underscore that combining defect engineering with rare‐earth doping offers a versatile strategy for designing stable, tunable materials for spintronic applications.

## Conflict of Interest

The authors declare no conflict of interest.

## Author Contributions

A.k.A., W.‐K.C., and M.‐H.W. contributed equally to this work. A.k.a., H.‐Y.T.C., A.L.W., and C.H.L. conceived the idea, designed the experiments, and initiated the theoretical calculations. M.‐H.W., F.‐C.H., A.C.G., and H.‐H.C. conducted the SQUID measurements, while M.C. and S.G. performed the Raman measurements. Material synthesis was carried out by M.‐H.W., F.‐C.H., and A.k.A. A.S. conducted the electron microscopy experiments. Synchrotron measurements were performed and coordinated by C.‐Y.C., H.‐M.T., H.‐J.L., and S.‐C.H. Theoretical calculations were carried out by H.‐Y.T.C. and W.‐K.C. The project was supervised by L.‐C.H., K.‐H.C., N.‐H.T., Y.‐L.C., S.‐Y.W., H.‐Y.T.C., A.L.W., and C.H.L. The manuscript was drafted and edited by A.k.A., W.‐K.C., M.‐H.W., H.‐Y.T.C., A.L.W., and C.H.L., with all authors participating in editing the final version.

## Supporting information



Supporting Information

## Data Availability

The data that support the findings of this study are available from the corresponding author upon reasonable request.
