# Supplementary material for: Revealing Robust Room Temperature Ferromagnetism in Gd‐Doped Few‐Layered MoS2 Thin Films
Source: Adv Sci (Weinh). 2025 Aug 29;12(43):e10366. doi: 10.1002/advs.202510366 (PMC12631849; doi:10.1002/advs.202510366)
Supplement: Supplementary file 1 — Supporting Information [file ADVS-12-e10366-s001.docx]

**Supporting information**

**Revealing Robust Room Temperature Ferromagnetism in Gd-Doped Few-Layered MoS_2_ Thin Films**

Aswin kumar Anbalagan ^1, 2, †, *^, Weng-Kent Chan ^3, †^, Ming-Hsuan Wu ^1, †^, Fang-Chi Hu ^1^, Hsin-Hao Chiu ^4^, Amr Sabbah ^5, 6^, Mayur Chaudhary ^7^, Shivam Gupta ^7^_,_ Kai-Wei Chuang ^8^, Ashish Chhaganlal Gandhi ^9, 10^, Ching-Yu Chiang ^11^, Huang-Ming Tsai ^11^, Shu-Chih Haw ^11^, Kirankumar Venkatesan Savunthari ^12^, Hong-Ji Lin ^11^, Li-Chyong Chen ^6, 13^, Kuei-Hsien Chen ^5, 6^, Nyan-Hwa Tai ^7^, Yu-Lun Chueh ^7^, Sheng-Yun Wu ^4^, Hsin-Yi Tiffany Chen ^1, 3, 7, *^ , Andrew L. Walter ^2, *^ and Chih-Hao Lee ^1, 8 , *^

^1^Department of Engineering and System Science, National Tsing Hua University, Hsinchu 300044, Taiwan

^2^National Synchrotron Light Source II, Brookhaven National Laboratory, Upton, New York 11973, USA

^3^College of Semiconductor Research, National Tsing Hua University, Hsinchu 300044, Taiwan

^4^Department of Physics, National Dong Hwa University, Hualien 97401, Taiwan

^5^Institute of Atomic and Molecular Sciences, Academia Sinica, Taipei 10617, Taiwan

^6^Center for Condensed Matter Sciences, National Taiwan University, Taipei 10617, Taiwan

^7^Department of Materials Science and Engineering, National Tsing Hua University, Hsinchu 300044, Taiwan

^8^Institute of Nuclear Engineering and Science, National Tsing Hua University, Hsinchu 300044, Taiwan

^9^Department of Electrical Engineering, National Tsing Hua University, Hsinchu 300044, Taiwan.

^10^Electrical and Computer Engineering, Lyle School of Engineering, Southern Methodist University, Dallas, TX 75205, USA

^11^National Synchrotron Radiation Research Center, Hsinchu 30076, Taiwan

^12^Department of Chemistry and Chemical Biology, Northeastern University, Boston, Massachusetts 02115, United States

^13^Center of Atomic Initiative for New Materials, National Taiwan University, Taipei 10617, Taiwan
**^†^**These authors contributed equally to this work.

^*^Correspondence: [aanbalaga1@bnl.gov](mailto:aanbalaga1@bnl.gov) (A.k.A.), [hsinyi.tiffany.chen@gapp.nthu.edu.tw](mailto:hsinyi.tiffany.chen@gapp.nthu.edu.tw) (H-Y.T.C.), [awalter@bnl.gov](mailto:awalter@bnl.gov) (A.L.W.), chlee@mx.nthu.edu.tw (C-H.L.)

1. **Experimental and Computational Method**

**1.1 Preparation of pristine and Gd-doped MoS_2_ thin films -** DC & RF magnetron sputtering techniques were employed to deposit the pristine MoS_2_ and Gd-doped MoS_2_ few-layered and bulk films onto a 1 cm x 1 cm Si wafer (100) containing a 300 nm thermally grown SiO_2_ layer on it. The sputtering chamber was maintained at a base pressure of about 2 x 10^-6^ Torr, and the sputtering was performed under a working pressure of 5 x 10^-3^ Torr in an Ar environment.

Pristine MoS_2_ films were sputtered by DC sputtering at 20 W and different thicknesses of MoS_2_ films (*i.e.* 3.5 nm and 40 nm) were achieved by varying the deposition time. Gd-doped MoS_2_ samples were prepared by co-sputtering, where the MoS_2_ target (99.99%) was sputtered at 20 W using DC power and the Gd target (99.99%) was sputtered using an RF sputtering gun at different sputtering powers such as: 8 W, 10 W, 12 W, and 20 W to obtain varying dopant concentrations.

**1.2 Characterization instruments -** Transmission electron microscopy (TEM) images were captured using a JEOL2100F transmission electron microscope. The cross-section of the MoS_2_ films were prepared through Focused Ion Beam Scanning Electron Microscopy (FIB-SEM) using the SEIKO SMI3050SE. For TEM analysis, samples were prepared with carbon and Pt as protective layers using the FIB method and then transferred onto a lacey copper grid to measure the thickness of few-layered and bulk Gd doped MoS_2_ films. DC magnetization measurements were performed with a superconducting quantum interference device magnetometer (VSM SQUID, Quantum Design, USA) at room temperature (RT). Raman spectroscopy using a 532 nm excitation laser (HORIBA HR800) was performed to investigate the variations in the chemical structure of the MoS_2_ films of different thicknesses before and after Gd doping. The variations in the chemical states of the Gd: MoS_2_ films were analyzed with an X-ray photoelectron spectroscopy (XPS) (ULVAC-PHI, PHI Quantera II) equipped with an Al−K_α_ excitation source. Synchrotron X-ray diffraction (XRD), X-ray magnetic circular dichroism (XMCD) and X-ray fluorescence (XRF) techniques were performed at beamline BL17B of the Taiwan Light Source (TLS), beamline BL21A of the Taiwan Photon Source (TPS) BL21A and, beamline 45A of the National Synchrotron Radiation Research Center (NSRRC) to study the crystal structure, microscopic magnetic properties and elemental uniformity, of the pristine and Gd doped MoS_2_ films. Scanning electron microscopy (JEOL, JSM-7610F) was used to study the uniformity, and morphology of the bulk MoS_2_ (40 nm) films before and after doping.

**1.3 Post annealing treatment -** To gain deeper insights into the impact of defects on different Gd doped MoS_2_ films, we conducted post-annealing treatments on the film exhibiting the highest saturation magnetization. These post annealing treatments were carried out under argon, vacuum and H_2_S environments for 15 min each, aiming to observe changes in both magnetic moment and chemical vibrations existing in the Gd doped MoS_2_ films.

**1.4 Density Functional Theory -** Spin-polarized density functional theory (DFT)^[1]^ calculations were carried out using the Vienna Ab initio Simulation Package (VASP)^[2]^. The Perdew–Burke–Ernzerhof (PBE)^[3]^ functional within the generalized gradient approximation (GGA) was employed to account for electron exchange and correlation effects. The projector-augmented wave (PAW) ^[4-5]^ with cut-off energy of 550 eV and Grimme’s dispersion correction (DFT-D3)^[6]^ was applied throughout the calculations. Structure optimizations were performed using a conjugate gradient algorithm until the force magnitudes were less than 0.01 eV Å^−1^ on all atoms and the energy difference between self-consistent electronic iterations was less than 10^−5^ eV. In this study, we modeled both pristine and Gd-doped defective bulk MoS_2_ using a 3 × 3 supercell dimensions (9.578 Å × 9.578 Å × 12.418 Å), whereas the monolayer MoS_2_ model was represented by a 4 × 4 supercell (12.770 Å × 12.770 Å × 33.273 Å with a 30 Å vacuum space included). For monolayer MoS_2_ (4 × 4 supercell), a Γ-centered 2 × 2 × 1 k-point mesh was used for structural optimization. For bulk MoS_2_ (3 × 3 supercell), a Γ-centered 3 × 3 × 2 k-point mesh was employed (the details structural parameters are illustrated in **Table S2**). To compute the magnetization and density of states, denser k-point meshes of 6 × 6 × 1 and 6 × 6 × 4 were used for the monolayer and bulk structures, respectively. The DFT + U method ^[7]^ is employed to calculate the on-site Coulomb correlation of the Gd 4f electrons where the U was set to 6.0 eV^[8]^. In addition, monolayer MoS_2_ (4 × 4 supercell) with S adatom and bulk MoS_2_ (4 × 4 supercell) with interstitial S atom were calculated to investigate the migration barrier. The transition states were located using a climbing image nudged elastic band method (CI-NEB)^[9]^.

The formation energy ($E_{form}$) for both monolayer and bulk Gd-doped and defective MoS_2_ was computed with the following equation:

$E_{form}=E_{defective(MoS_{2})}+ \left( n_{V\left( Mo \right)}+n_{Gd} \right)E_{Mo}+n_{v\left( S \right)}E_{S}- n_{Gd}E_{Gd}-E_{pristine(MoS_{2})}$

where $E_{form}$ is the formation energy of the MoS_2_ model, $E_{defective\left( MoS_{2} \right)}$ is the total energy of the Gd-doped MoS_2_ defective model, $n_{V(Mo)}$ is the number of Mo vacancies, $n_{Gd}$ is the number of Gd dopant atom, $E_{Mo}$is the energy of single Mo atom in the bulk cubic Mo model, $n_{V(S)}$ is the number of S vacancies, $E_{S}$ is the energy of single S atom in the bulk S orthorhombic model, $E_{Gd}$ is the energy of single Gd atom in the bulk hexagonal model and $E_{pristine(MoS_{2})}$ is the total energy of the MoS_2_ pristine model.

**
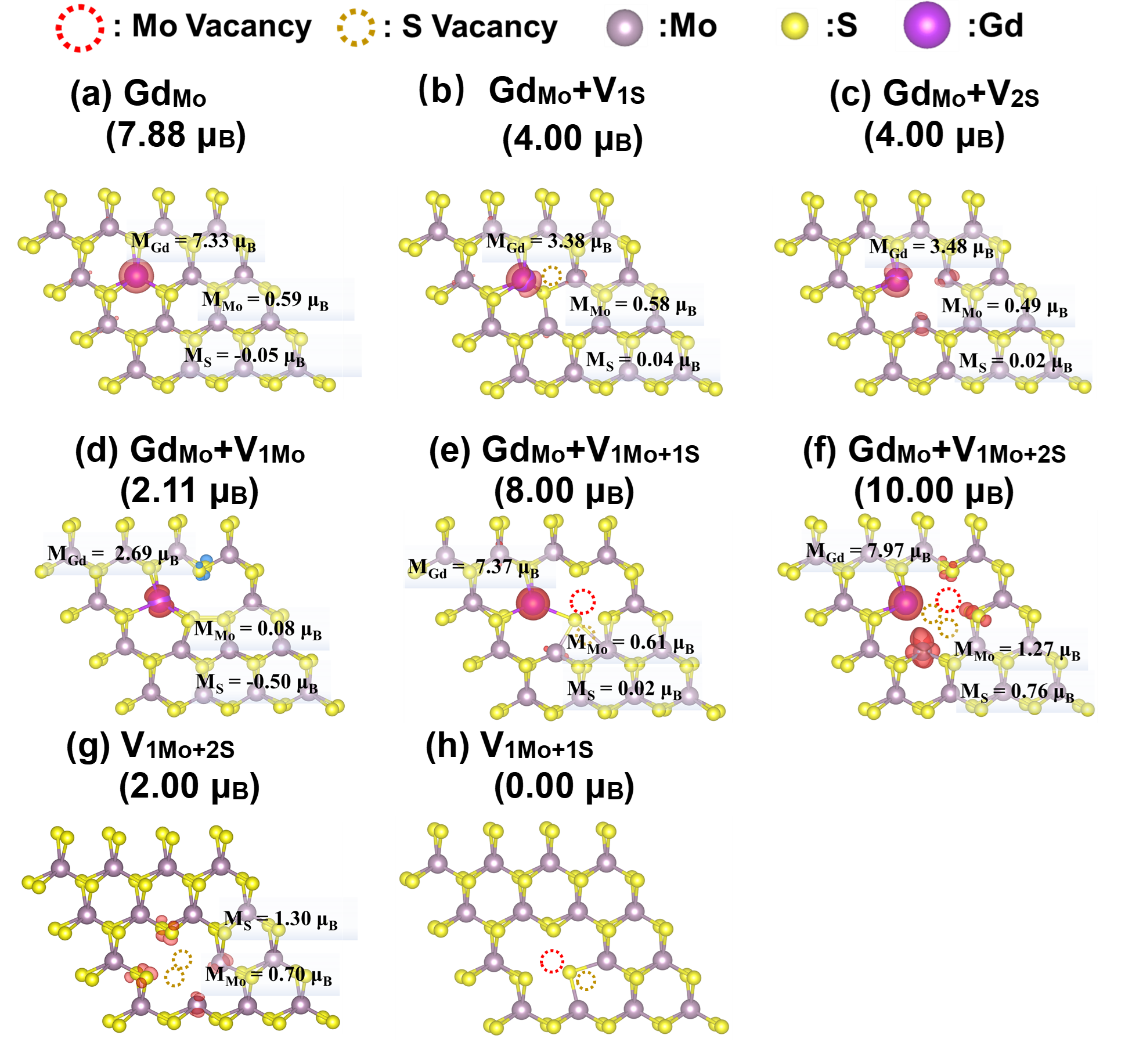
**

**Figure S1.** Spin density distribution of a 4 × 4 supercell monolayer MoS_2_ model. Red clouds represent spin-up density, and pink clouds represent spin-down density. The symbols indicate: Mo (grey), S (yellow), Gd (purple), Mo vacancy (dashed red circle), and S vacancy (dashed yellow circle).

**
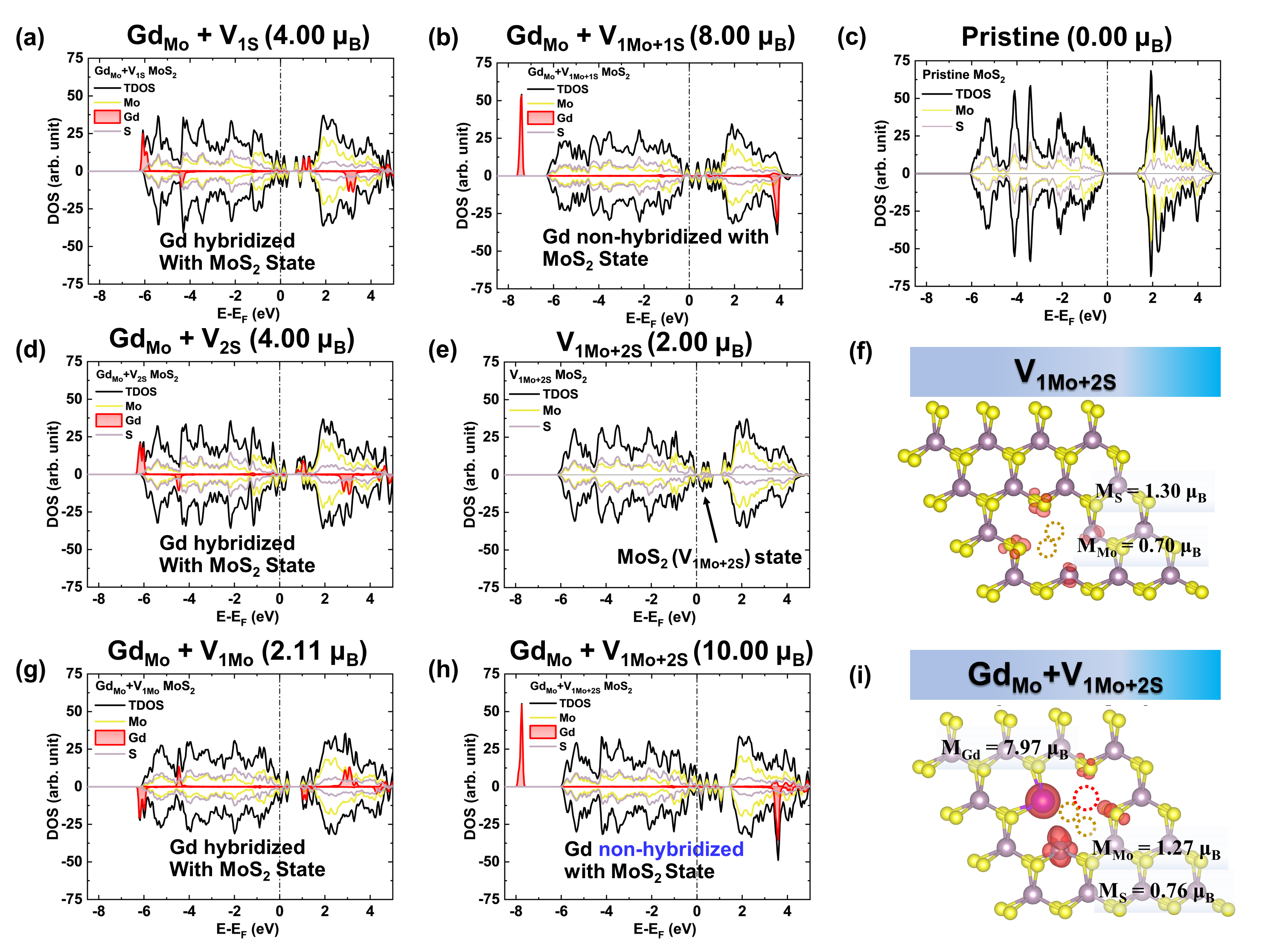
**

**Figure S2.** Total density of states (TDOS) and projected density of states (PDOS) for MoS_2_ models with various defect configurations. Panels show: (a) Gd_Mo_+V_1S_ , (b) Gd_Mo_+V_1Mo+1S_ , (c) pristine , (d) Gd_Mo_+V_2S_ , (e) V_1Mo+2S_ , (g) Gd_Mo_+V_1Mo_ , and (h) Gd_Mo_+V_1Mo+2S_ MoS_2_. Panels (f) and (i) show spin density distributions for V_1Mo+2S_ and Gd_Mo_+V_1Mo+2S_, respectively. Spin density maps illustrate spin-up density (red clouds) and spin-down density (pink clouds). Structural models identify defect sites: Mo (grey), S (yellow), Gd (purple), Mo vacancy (dashed red circle), and S vacancy (dashed yellow circle). Magnetic moments are indicated, underscoring localized magnetization near defect sites.

**
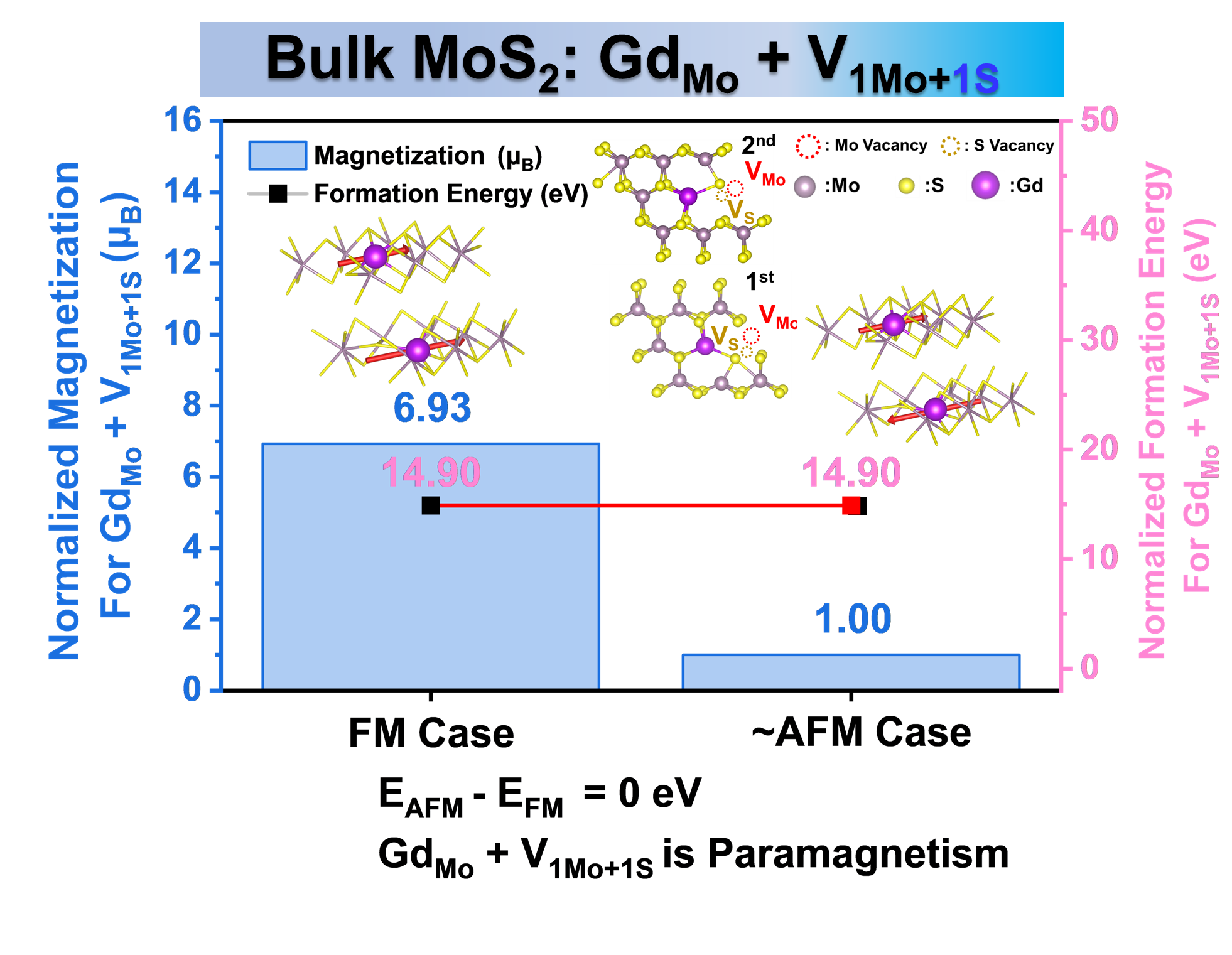
**

**Figure S3.** Normalized magnetization (μ_B_ per layer, left axis) and normalized formation energy (eV per layer, right axis) for the bulk MoS_2_ model featuring a Gd substitution at a Mo site (Gd_Mo_) and a single Mo and a single S vacancy (V_1Mo+1S_). The left and right structural figures show the ferromagnetic (FM) and antiferromagnetic (AFM) spin polarizations of the Gd atom within the defective bulk supercell. Antiferromagnetism was modeled by assigning opposite magnetization directions to the primary contributors, the Gd atoms, in bulk sample. The middle structural model shows the top view of bulk MoS_2_ with Gd_Mo_+V_1Mo+1S_ defect, where atom types and defect sites are depicted as: Mo (grey), S (yellow), Gd (purple), Mo vacancy (dashed red circle), and S vacancy (dashed yellow circle).


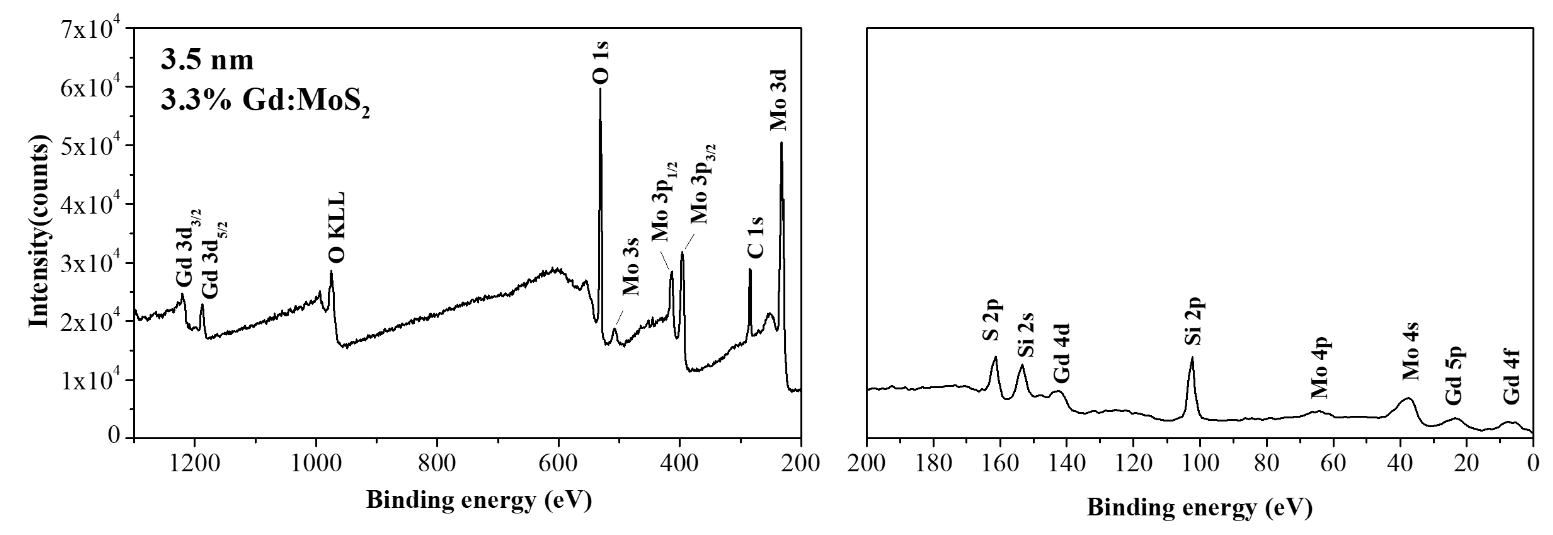


**Figure S4.** XPS survey spectra of 3.3 % Gd doped MoS_2_ few-layered (3.5 nm) film.

To determine the elemental composition and relative proportions of Gd, Mo, and S in the Gd-doped MoS₂ films, we carried out X-ray photoelectron spectroscopy (XPS) measurements. XPS provides several types of information based on the measured binding energies: identification of elements; insights into the chemical environment, including chemical shifts and bonding states; and quantitative analysis through atomic concentration estimates. The atomic concentration of an element can be estimated using a simple equation [20]:

I = n JσKλ= n SF (1)

where I is the intensity of a photoelectron peak from a homogeneous material, n is the atomic concentration of the atom or ion, J is the photon flux, σ is the cross-section for photoelectron production (which depends on the element and energy being considered), K is a term which covers all of the instrumental factors described above, λ is the electron attenuation length, and SF is the sensitive factor compose of σ, K, λ.

For the analysis, we applied Shirley background subtraction to remove background signals, and we performed peak fitting using a Voigt function (a convolution of Gaussian and Lorentzian components). XPS is a powerful technique not only for identifying elemental presence but also for estimating atomic concentrations based on the integrated peak areas and the relative sensitivity factors (RSFs) for each element. Using this method, we quantified the atomic concentrations of Gd, Mo, and S in our films. The RSF values and peak fitting parameters used for this analysis are presented in Tables S3 and S4. Our results show that the Gd doping concentration increases systematically with the RF power applied during the co-sputtering process. Specifically, the Gd atomic concentrations in the samples prepared at 8 W, 10 W, 12 W, and 20 W RF power were approximately 0.36%, 0.47%, 1.05%, and 3.3%, respectively.


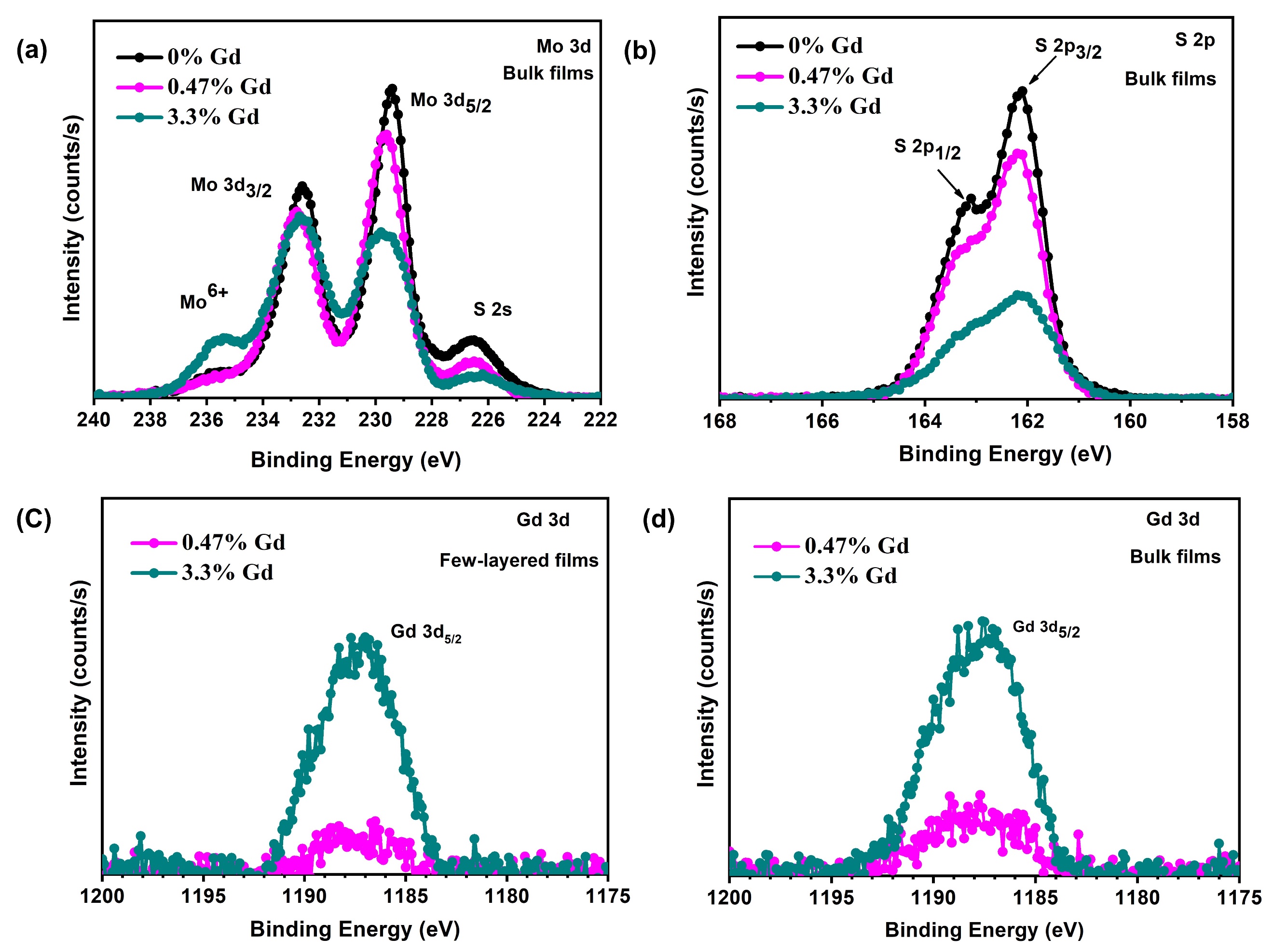


**Figure S5.** XPS spectra of (a) Mo 3d and (b) S2p of bulk (40 nm) Gd doped MoS_2_ films. and Gd 3d spectra of (c) few-layered (3.5 nm) and (d) 40 nm Gd doped MoS_2_ films.


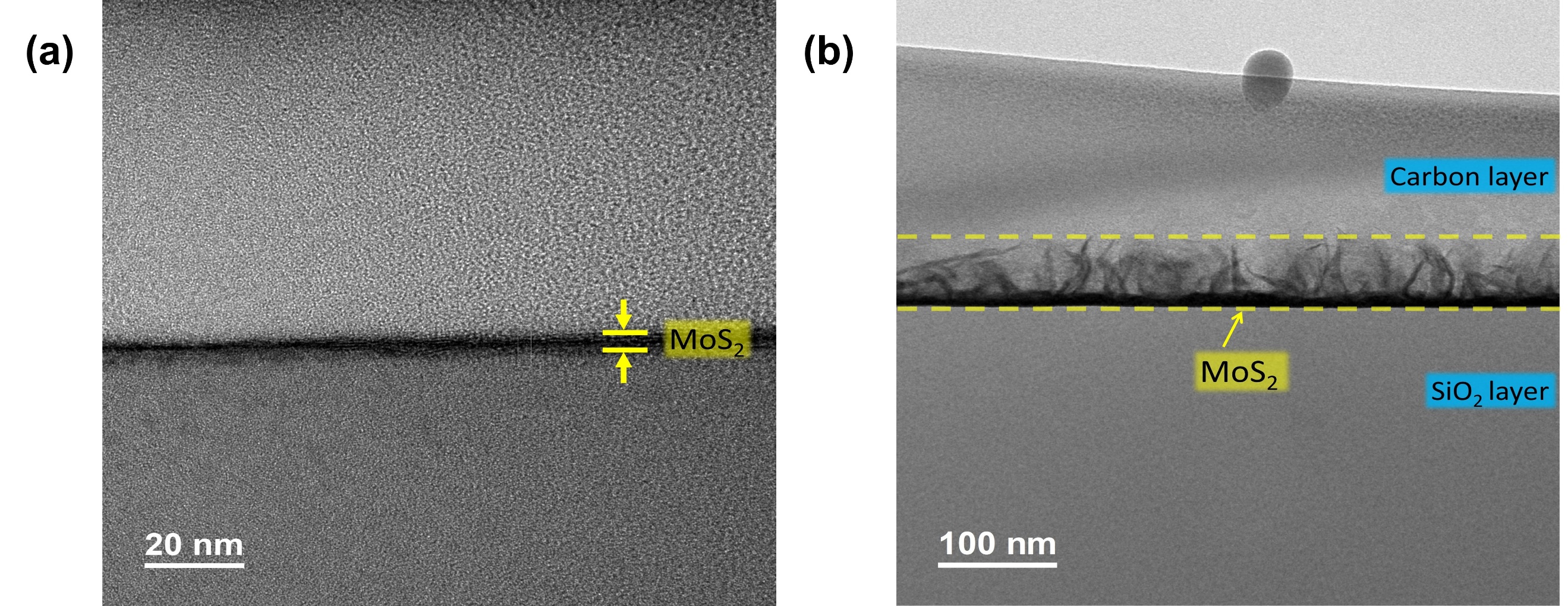


**Figure S6.** Cross-section TEM images of pristine MoS_2_ films on SiO_2_/Si substrate prepared by FIB cutting; a) few layered MoS_2_ film, and b) Bulk MoS_2_ film.


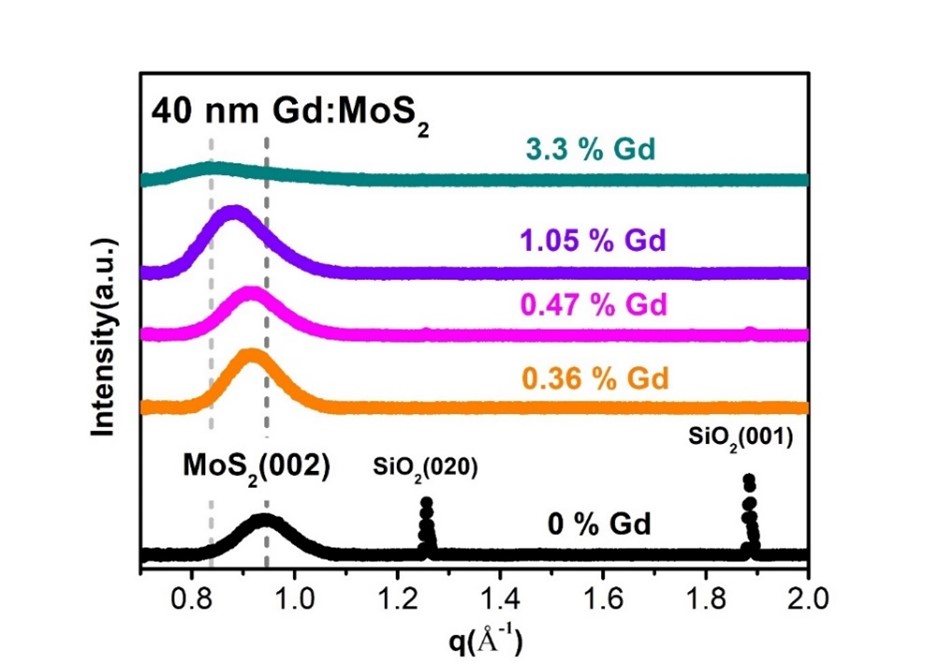


**Figure S7.** Plane-normal XRD pattern of 40 nm thick Gd: MoS_2_ films with different Gd dopant concentration. And the JCPDS No. for MoS_2_ is (37-1492) and standard pattern of zeolite -phase SiO_2_ (ICSD No. 98-009-3715).

XRF results, presented in **Figure S8,** include mapping for samples with 3.3 % Gd doping in both 3.5 nm and 40 nm films, representing the highest Gd doping concentrations studied across different thicknesses. XRF mapping is a bulk-sensitive characterization technique that offers statistically meaningful insights into elemental distributions over large areas. Our mapping was performed across two scan sizes: a larger scan area of 50 μm × 50 μm area and a high-resolution scan of 5 μm× 5 μm scan area, both with a pixel resolution of 0.1 μm × 0.1 μm. This multi-scale approach allows us to assess elemental homogeneity at both macroscopic and sub-micron levels. In both scan regions, the intensity variations for Gd, Mo, and S were minimal, suggesting a **uniform distribution of Gd dopants and sulfur vacancies** throughout the films. We did not observe any signs of Gd clustering or surface segregation within the resolution limits (~100 nm), making it unlikely that the observed high magnetization arises from large Gd-rich agglomerates.


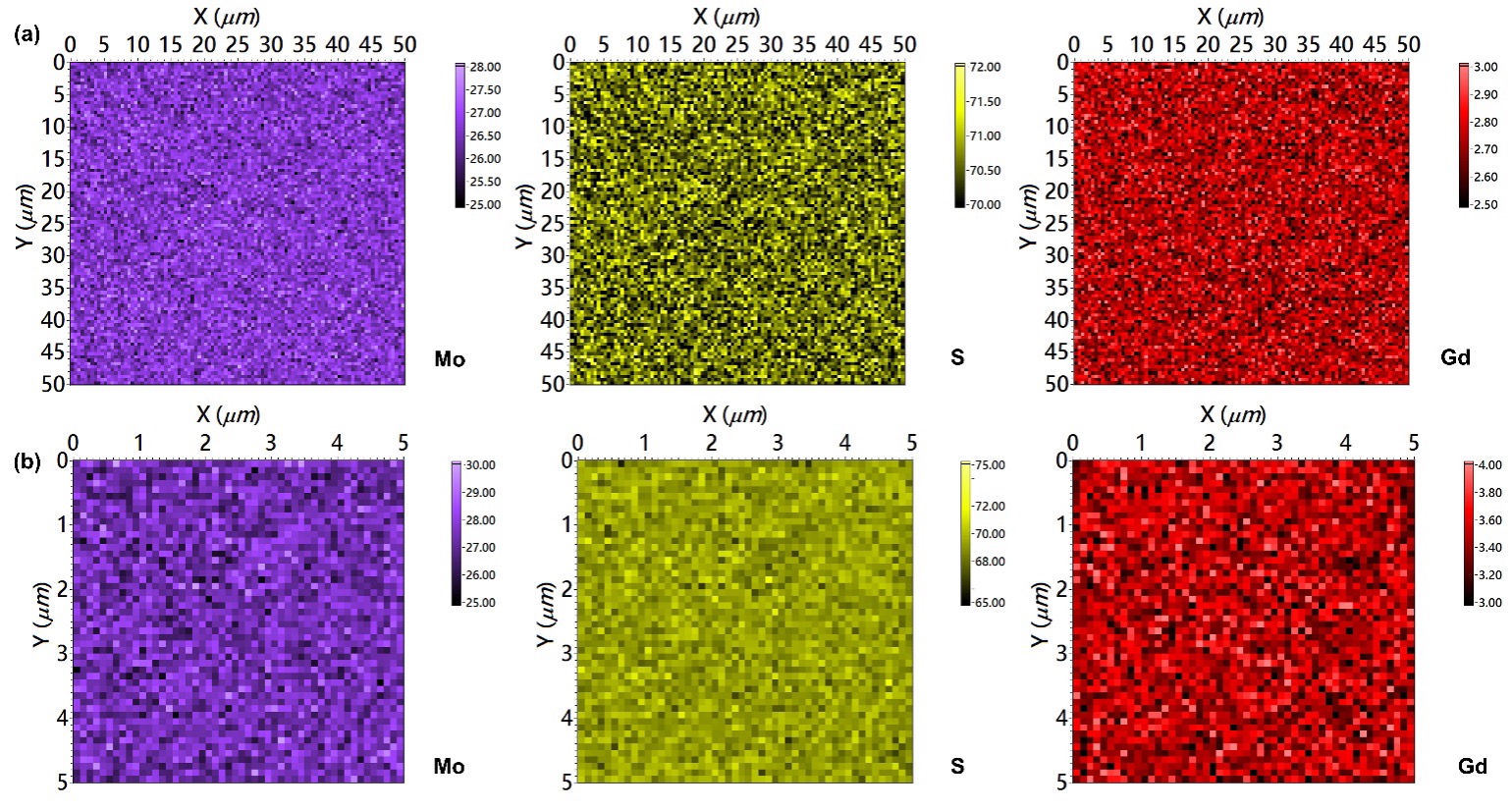


**Figure S8.** XRF mapping of 3.3 % Gd: MoS_2_ films: (a) few-layered (3.5 nm) and (b) bulk (40 nm). Yellow represents S atoms, purple represents Mo atoms and red represents Gd atoms. The scale bar represents the intensity of presence of individual elements in the sample.


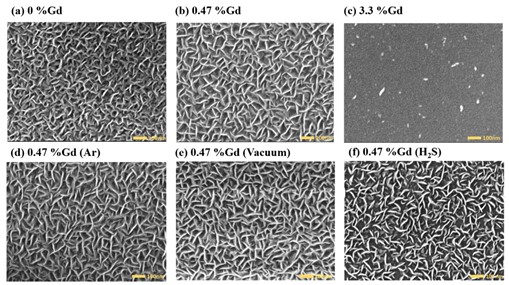


**Figure S9.** SEM morphology of: (a)Pristine, (b) 0.47 % and (c) 3.3 % Gd doped MoS_2_ (40 nm) bulk films.

**Table S1.** Comparison of saturation magnetization (M_s_) values from this work with other previous published works

| **Dopants used** | **Form of MoS_2_** | **Thickness** | **M_s_ value** | **Ref** |
| --- | --- | --- | --- | --- |
| Cu-doped | Polycrystalline | Nanosheets | 0.023 emu/g | [10] |
| Mn-doped | Polycrystalline | 10 layers | 0.014 emu/g | [11] |
| Cr-doped | Polycrystalline | 2 mm | 0.016 emu/g | [12] |
| Re-doped | Polycrystalline | Nanosheets | 0.008 emu/g | [13] |
| Dy-doped | Polycrystalline | Nanosheets | 0.003 emu/g | [14] |
| Ho-doped | Polycrystalline | NA | 0.055 emu/g | [15] |
| Mn-doped | Single crystal | NA | 125 emu/cm^­3^ | [16] |
| Fe-doped | Single crystal | NA | 15 emu/cm^­3^ | [16] |
| Ni-doped | Single crystal | NA | 4 emu/cm^­3^ | [16] |
| Co-doped | Single crystal | NA | 40 emu/cm^­3^ | [16] |
| Nd-doped | Single crystal | NA | 50 emu/cm^­3^ | [17] |
| Co/Nb-doped | Single crystal | NA | 15 emu/cm^­3^ | [18] |
| Co/Nd-doped | Single crystal | NA | 80 emu/cm^­3^ | [19] |
| Gd-doped | Polycrystalline | 3.5 nm | 454 emu/cm^­3^ | This work* |

| **Table S2.** The structure with their supercell, defect types, dimensions (a, b, and c in Å), and composition (Mo, Gd, and S atoms) along with total atom counts for monolayer (4×4) and bulk (3×3) MoS_2_ supercells for figure 2. For bulk models, defects are present in both layers. | | | | | | | | |
| --- | --- | --- | --- | --- | --- | --- | --- | --- |
| **Structure** | **Defect**  **Type** | **a**  **(Å)** | **b**  **(Å)** | **c**  **(Å)** | **Mo atom** | **Gd atom** | **S**  **atom** | **Total number of atoms** |
| Monolayer  (4×4 supercell) | Gd_Mo_ | 12.770 | 12.770 | 33.273 | 15 | 1 | 32 | 48 |
|  | Gd_Mo_ + V_1S_ |  |  |  | 15 | 1 | 31 | 47 |
|  | Gd_Mo_ + V_2S_ |  |  |  | 15 | 1 | 30 | 46 |
|  | Gd_Mo_ + V_1Mo_ |  |  |  | 14 | 1 | 32 | 47 |
|  | Gd_Mo_ + V_1Mo+1S_ |  |  |  | 14 | 1 | 31 | 46 |
|  | Gd_Mo_ + V_1Mo+2S_ |  |  |  | 14 | 1 | 30 | 45 |
|  | V_1Mo+2S_ |  |  |  | 15 | 0 | 30 | 45 |
|  | Pristine |  |  |  | 16 | 0 | 32 | 48 |
| Bulk  (3×3 supercell) | Gd_Mo_ | 9.578 | 9.578 | 12.418 | 16 | 2 | 36 | 54 |
|  | Gd_Mo_ + V_1S_ |  |  |  | 16 | 2 | 34 | 52 |
|  | Gd_Mo_ + V_2S_ |  |  |  | 16 | 2 | 32 | 50 |
|  | Gd_Mo_ + V_1Mo_ |  |  |  | 14 | 2 | 36 | 52 |
|  | Gd_Mo_ + V_1Mo+1S_ |  |  |  | 14 | 2 | 34 | 50 |
|  | Gd_Mo_ + V_1Mo+2S_ |  |  |  | 14 | 2 | 32 | 48 |
|  | Pristine |  |  |  | 18 | 0 | 36 | 54 |

**Table S3**. XPS Quantification results of few-layered (3.5 nm) pristine and Gd-doped MoS_2_ films.

| Gd conc. | Elements | Peak area | Peak area/R.S.F | | At. ratio | At. Conc. (%) |
| --- | --- | --- | --- | --- | --- | --- |
| 0 % | Mo 3d_5/2_ | 15361.40 | 129.45 | 1.00 | | 31.49% |
|  | S 2p | 11217.30 | 281.62 | 2.18 | | 68.51% |
| 0.36 % | Gd 3d_5/2_ | 433.30 | 0.91 | 0.01 | | 0.36% |
|  | Mo 3d_5/2_ | 9849.40 | 83.00 | 1.00 | | 32.94% |
|  | S 2p | 6695.00 | 168.09 | 2.03 | | 66.70% |
| 0.47 % | Gd 3d_5/2_ | 505.00 | 1.06 | 0.01 | | 0.46% |
|  | Mo 3d_5/2_ | 9331.40 | 78.63 | 1.00 | | 33.81% |
|  | S 2p | 6090.00 | 152.90 | 1.94 | | 65.74% |
| 1.05 % | Gd 3d_5/2_ | 1093.50 | 2.29 | 0.03 | | 1.06% |
|  | Mo 3d_5/2_ | 9009.40 | 75.92 | 1.00 | | 35.05% |
|  | S 2p | 5513.00 | 138.41 | 1.82 | | 63.89% |
| 3.3 % | Gd 3d_5/2_ | 2816.50 | 5.91 | 0.08 | | 3.31% |
|  | Mo 3d_5/2_ | 8473.30 | 71.40 | 1.00 | | 41.18% |
|  | S 2p | 3828.00 | 96.11 | 1.35 | | 55.42% |

**Table S4**. XPS Quantification results of bulk (40 nm) pristine and Gd doped MoS_2_ films.

| Gd conc. | Elements | Peak area | Peak area/R.S.F | | At. ratio | At. Conc.  (%) |
| --- | --- | --- | --- | --- | --- | --- |
| 0 % | Mo 3d_5/2_ | 16369.00 | 137.94 | 1.00 | | 31.33% |
|  | S 2p | 12044.20 | 302.38 | 2.19 | | 68.67% |
| 0.36 % | Gd 3d_5/2_ | 662.00 | 1.39 | 0.01 | | 0.37% |
|  | Mo 3d_5/2_ | 14492.00 | 122.12 | 1.00 | | 32.61% |
|  | S 2p | 9999.00 | 251.04 | 2.06 | | 67.02% |
| 0.47 % | Gd 3d_5/2_ | 828.50 | 1.74 | 0.01 | | 0.48% |
|  | Mo 3d_5/2_ | 14252.40 | 120.10 | 1.00 | | 33.40% |
|  | S 2p | 9468.00 | 237.70 | 1.98 | | 66.11% |
| 1.05 % | Gd 3d_5/2_ | 1585.20 | 3.32 | 0.03 | | 1.05% |
|  | Mo 3d_5/2_ | 13011.60 | 109.65 | 1.00 | | 34.73% |
|  | S 2p | 8075.00 | 202.73 | 1.85 | | 64.22% |
| 3.3 % | Gd 3d_5/2_ | 3426.30 | 7.18 | 0.08 | | 3.34% |
|  | Mo 3d_5/2_ | 10391.50 | 87.57 | 1.00 | | 40.71% |
|  | S 2p | 4793.00 | 120.33 | 1.37 | | 55.95% |

**References**

[1] P. Hohenberg, W. Kohn, *Inhomogeneous Electron Gas*, *Phys. Rev.* **1964**, *136* (3B), B864

[2] G. Kresse, J. Furthmüller, *Efficiency of ab-initio total energy calculations for metals and semiconductors using a plane-wave basis set*, *Comput. Mater. Sci.* **1996**, *6* (1), 15

[3] J. P. Perdew, K. Burke, M. Ernzerhof, *Generalized Gradient Approximation Made Simple*, *Phys. Rev. Lett.* **1996**, *77* (18), 3865

[4] P. E. Blöchl, *Projector augmented-wave method*, *Phys. Rev. B.* **1994**, *50* (24), 17953

[5] G. Kresse, D. Joubert, *From ultrasoft pseudopotentials to the projector augmented-wave method*, *Phys. Rev. B.* **1999**, *59* (3), 1758

[6] S. Grimme, *Semiempirical GGA-type density functional constructed with a long-range dispersion correction*, *J. Comput. Chem.* **2006**, *27* (15), 1787

[7] O. Bengone, M. Alouani, P. Blöchl, J. Hugel, *Implementation of the projector augmented-wave LDA+U method: Application to the electronic structure of NiO*, *Phys. Rev. B.* **2000**, *62* (24), 16392

[8] C. N. M. Ouma, S. Singh, K. O. Obodo, G. O. Amolo, A. H. Romero, *Controlling the magnetic and optical responses of a MoS_2_ monolayer by lanthanide substitutional doping: a first-principles study*, *Phys. Chem. Chem. Phys.* **2017**, *19* (37), 25555

[9] G. Henkelman, B. P. Uberuaga, H. Jónsson, *A climbing image nudged elastic band method for finding saddle points and minimum energy paths*, *J. Chem. Phys.* **2000**, *113* (22), 9901

[10] B. Xia, Q. Guo, D. Gao, S. Shi, K. Tao, *High temperature ferromagnetism in Cu-doped MoS_2_ nanosheets*, *J. Phys. D Appl. Phys.* **2016**, *49* (16), 165003

[11] J. Wang, F. Sun, S. Yang, Y. Li, C. Zhao, M. Xu, Y. Zhang, H. Zeng, *Robust ferromagnetism in Mn-doped MoS_2_ nanostructures*, *Appl. Phys. Lett.* **2016**, *109* (9), 092401

[12] R. Zhang, Y. Du, G. Han, X. Gao, *Ferromagnetism and microwave absorption properties of Cr-doped MoS_2_ nanosheets*, *J. Mater. Sci.* **2019**, *54* (1), 552

[13] B. Xia, P. Liu, Y. Liu, D. Gao, D. Xue, J. Ding, *Re doping induced 2H-1T phase transformation and ferromagnetism in MoS_2_ nanosheets*, *Appl. Phys. Lett.* **2018**, *113* (1), 013101

[14] Q. Zhao, Q. Lu, Y. Liu, M. Zhang, *Two-dimensional Dy doped MoS_2_ ferromagnetic sheets*, *Appl. Surf. Sci.* **2019**, *471*, 118

[15] Q. Zhao, C. Zhai, Q. Lu, M. Zhang, *Effect of Ho dopant on the ferromagnetic characteristics of MoS_2_ nanocrystals*, *Phys. Chem. Chem. Phys.* **2018**, *21* (1), 232

[16] Y. Wang, L.-T. Tseng, P. P. Murmu, N. Bao, J. Kennedy, M. Ionesc, J. Ding, K. Suzuki, S. Li, J. Yi, *Defects engineering induced room temperature ferromagnetism in transition metal doped MoS_2_*, *Mater. Des.* **2017**, *121*, 77

[17] X. Ding, X. Cui, A. Sohail, P. P. Murmu, J. Kennedy, N. Bao, J. Ding, R. Liu, M. Peng, L. Wang, X. Chu, A. Vinu, S. P. Ringer, J. Yi, *Defects Engineering Induced Ultrahigh Magnetization in Rare Earth Element Nd-doped MoS_2_*, *Adv. Quantum Technol.* **2021**, *4* (2), 2000093

[18] S. Ahmed, X.-Y. Carl Cui, X. Ding, P. P. Murmu, N. Bao, X. Geng, S. Xi, R. Liu, J. Kennedy, T. Wu, L. Wang, K. Suzuki, J. Ding, X. Chu, S. R. Clastinrusselraj Indirathankam, M. Peng, A. Vinu, S. P. Ringer, J. Yi, *Colossal Magnetization and Giant Coercivity in Ion-Implanted (Nb and Co) MoS_2_ Crystals*, *ACS Appl Mater Interfaces.* **2020**, *12* (52), 58140

[19] S. Ahmed, P. P. Murmu, C. I. Sathish, X. Guan, R. Geng, N. Bao, R. Liu, J. Kennedy, J. Ding, M. Peng, A. Vinu, J. Yi, *Co- and Nd-Codoping-Induced High Magnetization in Layered MoS_2_ Crystals*, *pss RRL* **2023**, *17* (6), 2200348

[20] The Electron Spectrum: Qualitative and Quantitative Interpretation, in An Introduction to Surface Analysis by XPS and AES. 2003. p. 59-77.
